# Impact of Fluoroalkylation on the n-Type Charge Transport of Two Naphthodithiophene Diimide Derivatives

**DOI:** 10.3390/molecules26144119

**Published:** 2021-07-06

**Authors:** Gaetano Ricci, Sofia Canola, Yasi Dai, Daniele Fazzi, Fabrizia Negri

**Affiliations:** 1 Dipartimento di Chimica “Giacomo Ciamician”, Università di Bologna, Via F. Selmi, 2, 40126 Bologna, Italy; gaetano.ricci@unamur.be (G.R.); canola@fzu.cz (S.C.); yasi.dai2@unibo.it (Y.D.); 2 Institut für Physikalische Chemie, Department für Chemie, Universität zu Köln, Greinstr. 4-6, D-50939 Köln, Germany; 3 INSTM, UdR Bologna, Via F. Selmi, 2, 40126 Bologna, Italy

**Keywords:** n-type, organic semiconductors, charge transport, organic crystals, charge mobility anisotropy, charge transfer, quantum chemistry, DFT, dynamic disorder, electron-phonon coupling

## Abstract

In this work, we investigate two recently synthesized naphthodithiophene diimide (NDTI) derivatives featuring promising n-type charge transport properties. We analyze the charge transport pathways and model charge mobility with the non-adiabatic hopping mechanism using the Marcus-Levich-Jortner rate constant formulation, highlighting the role of fluoroalkylated substitution in α (**α-NDTI**) and at the imide nitrogen (**N-NDTI**) position. In contrast with the experimental results, similar charge mobilities are computed for the two derivatives. However, while **α-NDTI** displays remarkably anisotropic mobilities with an almost one-dimensional directionality, **N-NDTI** sustains a more isotropic charge percolation pattern. We propose that the strong anisotropic charge transport character of **α-NDTI** is responsible for the modest measured charge mobility. In addition, when the role of thermally induced transfer integral fluctuations is investigated, the computed electron–phonon couplings for intermolecular sliding modes indicate that dynamic disorder effects are also more detrimental for the charge transport of **α-NDTI** than **N-NDTI**. The lower observed mobility of **α-NDTI** is therefore rationalized in terms of a prominent anisotropic character of the charge percolation pathways, with the additional contribution of dynamic disorder effects.

## 1. Introduction

Small molecule and polymeric n-type organic semiconductors are of great significance for the development of p–n diodes, organic field effect transistors (OFETs), thermoelectric organic devices, complementary circuits, and other plastic electronic devices [1,2,3,4,5,6]. Among the promising electron-deficient π-building blocks, dithienocoronenediimide [7], isoindigo derivatives [8], perylene diimide derivatives (PDI), [9,10,11,12,13] and diketopyrrolopyrrole [14,15] have been reported. Naphthalene diimide (NDI) was reported in 2000 [16] as a high-performance n-type semiconductor, and since then, several NDI derivatives have been designed to develop materials for dyes, pigments, sensors, and optoelectronic applications [13,17,18,19,20,21,22]. While core-linked NDI derivatives enabled the first high-mobility electron transport in polymer-based thin film transistors [19,23], a recently emerging class of materials consists of core-extended NDIs that afford rigid π frameworks with distinct molecular and electronic structures compared with the smaller NDIs [24]. Specifically, the planar and rigid structure over the whole π framework in core-extended acene and heteroacene diimides such as naphthodithiophene diimides (NDTI) can be beneficial for carrier transport, owing to smaller reorganization energies and enhanced intermolecular π-π overlap in the solid state [18,25,26]. Furthermore, substitution at the thiophene α positions in the NDTI core enabled a variety of NDTI derivatives [27,28,29,30], some of which displayed superior n-channel conduction in thin-film transistors of up to 0.73 cm^2^ V^−1^ s^−1^ by introducing chlorine groups into α positions [27,28].

To the best of our knowledge, there have been limited computational investigations concerning the charge transport properties of NDTI derivatives. In a recent study [31], the effects of electron-withdrawing groups and electron-donating substituents in the α position of NDTI derivatives were computationally investigated to establish design rules for novel semiconductors with both high charge transport properties and environmental stability. In some cases, however, crystal structures were not available, and the study was carried out under the assumption of a one-dimensional (1D) character of the charge transport.

**Figure 1:**
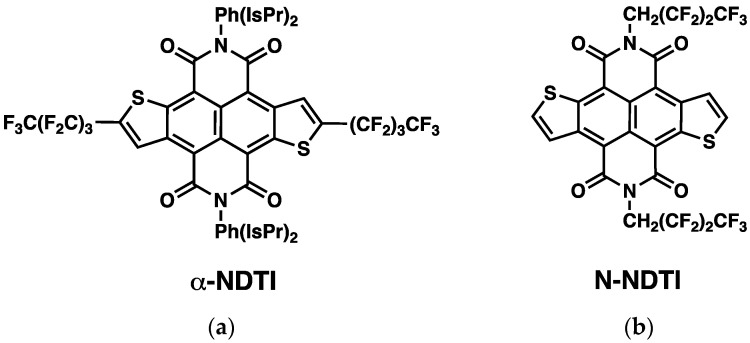
Structural formula of the two fluoroalkylated NDTI derivatives considered in this work. (**a**) **α-NDTI**. (**b**) **N-NDTI**.

The availability of organic single crystals, because of their general purity, low degree of defects and long-range order, is ideal for analyzing the intrinsic properties of the semiconductor as a function of the molecular arrangement. In this study, two recently synthesized fluoroalkyl-modified NDTI were considered (see Figure 1). One derivative featured fluoroalkyl substituents in the α position of the terminal thiophenes, while the second featured fluoroalkyl substituents on the N position and are hereafter labeled as **α-NDTI** (Figure 1a) and **N-NDTI** (Figure 1b), respectively. The introduction of fluorinated substituents is well known to improve the device stability under ambient conditions, but it also impacts the molecular packing arrangements, thereby leading to different device performances. Indeed, single-crystal OFETs of both fluoroalkyl-modified NDTI showed good electron transport properties, with **α-NDTI** displaying an average electron mobility of 0.037 cm^2^ V^−1^ s^−1^ and a highest value of 0.065 cm^2^ V^−1^ s^−1^, while **N-NDTI** scored an average value of 1.27 cm^2^ V^−1^ s^−1^ (highest value = 1.59 cm^2^ V^−1^ s^−1^) [32].

To model the charge transport properties of the two NDTI derivatives, we employed the non-adiabatic hopping approach recently adopted by us to rationalize the trends in n-type charge transport of various fluorinated and chlorinated PDI derivatives [33,34], as well as the anisotropy of charge transport in fluoroalkylated NDIs [35]. The integrated approach encompasses the quantum chemical (QC) evaluation of the electronic couplings and reorganization energies, followed by the calculation of charge transfer rate constants with different formulations [36]. The latter are then injected in kinetic Monte Carlo (KMC) simulations to model the charge percolations and mobilities. Dynamic disorder effects have been shown to influence the charge mobilities of organic semiconductors by inducing large fluctuations in the transfer integrals driven by slow intermolecular modes [37,38,39,40,41,42]. In previous investigations, we already considered thermally induced dynamical disorder effects [33,34,35,43], showing that for core unsubstituted PDI derivatives, the remarkable modulation of selected electronic couplings contributes to rationalizing the magnitude and directionality of the measured electron mobilities. Here, we compare the intra- and intermolecular parameters governing charge transport for the two NDTI derivatives, highlighting the impact of charge mobility anisotropy and eventually the role of dynamical disorder effects in affecting the charge mobility.

## 2. Results and Discussion

The crystal structures of **α-** and **N-NDTI** derivatives contain a certain degree of crystallographic disorder. More precisely, there are two possible orientations of thiophene rings inside the crystal, leaving the rest of the molecule identical in the two cases. For both NDTI derivatives, the dominant orientation was labeled *ANTI-1*, and the other was labeled *ANTI-2* (Figure S1). Both were considered in the calculation of electronic couplings and charge mobilities. To evaluate the effect of different thiophene orientations in the same solid phase, a crystal containing a mixture of the two orientations was also considered for **N-NDTI** (hereafter labeled *mix*). After single-molecule geometry optimization, the two **N-NDTI** structures corresponding to different thiophene orientations converged, as expected, to the same equilibrium geometry. In contrast, two slightly different optimized structures of **α-NDTI** *ANTI-1* and **α-NDTI** *ANTI-2* were obtained because of different conformers of the fluoroalkyl chains.

At their respective equilibrium structures, the lowest unoccupied molecular orbital (LUMO) energies of the two compounds (Table S1 and Figures S2 and S3) were low (−3.9 eV for **α-NDTI** and −3.8 eV for **N-NDTI)**, in line with previous calculations on NDTI derivatives [24,27,31] and with the requirements necessary to prevent the electron polaron from reducing ambient species [44]. In this regard, **α-NDTI** should be slightly favored over **N-NDTI**, since it displays a slightly lower LUMO energy.

### 2.1. Electronic Couplings and Charge Transfer Pathways

**Figure 2:**
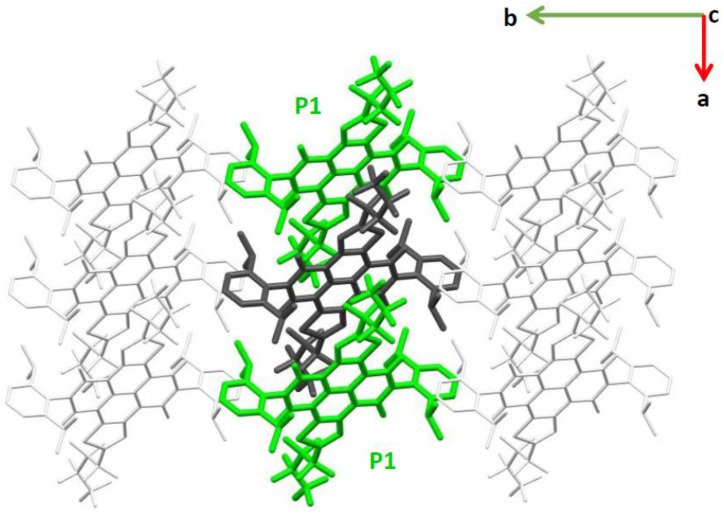
The most relevant charge transport path (P1, from the central dark gray to the lateral green molecules), shown within a portion of the **α-NDTI** crystal. The view is along the *c* axis.

The crystal of the **α-NDTI** display slipped one-dimensional (1D) stacking of its molecules (Figure 2 and Figures S4 and S5). The identification of all possible near neighbors of a given molecular site and the correspondingly computed charge transfer integrals (see Section 3) showed that only pathway P1, directed along the *a* axis (Figure 2), carried a significant electronic coupling, whereas all the others displayed negligible values (see Table 1 and Table S3). This is because P1 corresponds to the only molecular dimer in which intermolecular distance and π stacking allow for an efficient overlap between LUMO orbitals. Indeed, for **α-NDTI**, the combined effect of n and α substituents determined the solid phase packing with a markedly 1D columnar arrangement of the molecules. The electronic coupling associated with path P1, evaluated for *ANTI-1* and *ANTI-2*, displayed slightly varying values in the range of 36–40 meV (see Table 1).

**Figure 3:**
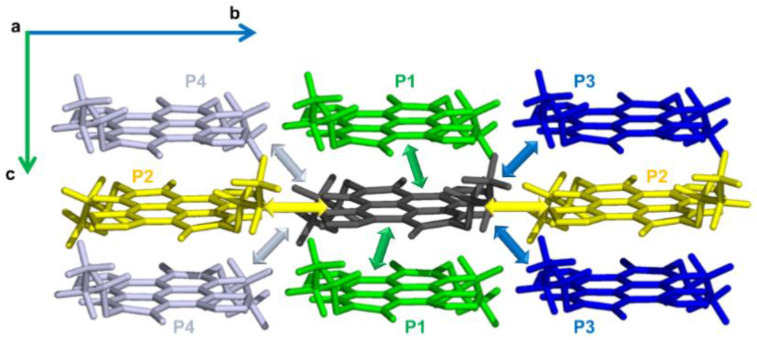
The most relevant charge transport paths (from the central dark gray molecule), namely P1 (green), P2 (yellow), P3 (blue), and P4 (light gray), shown within a portion of the **N-NDTI** crystal. The view is along the *a* axis.

The **N-NDTI** crystal was characterized by layers of molecules oriented with their conjugated plane almost perpendicular (edge-on) to the *b,c* plane (see Figure 3 and Figure S6). The molecules in each layer alternate two different site types that determine a twisted cofacial stacking. Notably, the packing between columns of π-stacked molecules, determined by the reduced number of substituents in this case, afforded smaller distances compared with **α-NDTI**. Because of the large distance between layers along the *a* direction, the most relevant paths for charge transport involved molecules in the same crystal layer (parallel to the *b,c* plane; see Figure 3), while negligible couplings were computed along the *a* direction. Electronic coupling calculations revealed three main paths that were expected to contribute significantly to charge transport (Table 1 and Figure 3). The P1 path defined a columnar percolation channel along the *c* axis between pairs of twisted molecules, with a coupling of 35 meV for **N-NDTI** *ANTI-1* and 50 meV for *ANTI-2*. The relative twisting of the two molecules forming each P1 dimer led to efficient π stacking with a small intermolecular and interplanar distance, resulting in efficient LUMO overlapping and significant electronic coupling.

Path P2 involved molecules belonging to the same site type, (therefore having the same spatial orientation) with a coupling of 10 meV (8 meV for *ANTI-2*). Because it was directed along the *b* axis, it would contribute to making charge transport more isotropic. Finally, path P3 defined a zig-zag percolation across two different columns of π-stacked molecules with a minor coupling of 2 meV (1 meV), and it would contribute to seldom jumps to adjacent columns. The larger P1 coupling for *ANTI-2* could be rationalized by a more efficient overlap between LUMO orbitals in the thiophene region (see Figure S7). In contrast, the overlap efficiency was slightly reduced for P2 and P3 in *ANTI-2*.

For the *mix* crystal of **N-NDTI** (see Figure S8), the P1 and P2 dimers were composed of one molecule featuring *ANTI-1* and one featuring *ANTI-2* thiophene orientations. In this case, because of different centers of mass, two different couplings of 46 and 38 meV were obtained for the two components of the P1 path (see Table 1), while the couplings were 9 and 8 meV for P2. In both cases, these were values within the range of those computed for the dimers formed by two identical molecules (*ANTI-1* or *ANTI-2*).

To summarize, fluoroalkyl substitution strongly impacted the solid state organization of the two NDTI derivatives and resulted in charge transfer integrals favoring a 1d charge percolation in **α-NDTI** and a more isotropic charge transfer network in **N-NDTI**. Charge transfer rate constants (Equation (4)) are, however, dependent not only on electronic couplings but also on reorganization energies, as is discussed in the next section.

### 2.2. Reorganization Energies

**Figure 4:**
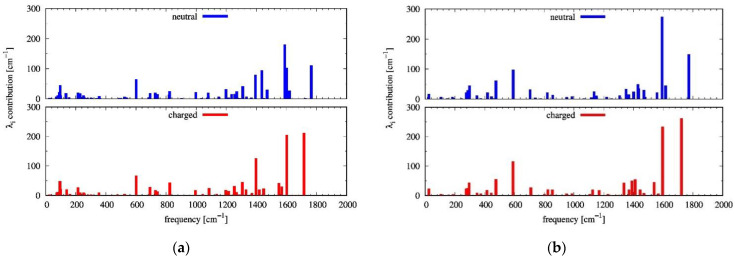
The vibrational frequency contributions (from HR factors, B3LYP/6-31G* level of theory) to the computed intramolecular reorganization energies $\lambda_{i}$ (for n-type charge transport) of (**a**) **α-NDTI** and (**b**) **N-NDTI**. Each graph shows the contribution from neutral species in the top part (blue bars) and from the negatively charged species in the bottom part (red bars).

The intramolecular reorganization energies computed using the four-point adiabatic potential (AP) method (see Tables S5 and S6) and from the computed Huang–Rhys (HR) factors $S_{m}$ (Table S7) agreed very closely (0.319 eV for **α-NDTI** and 0.305 eV for **N-NDTI**), which implied that the harmonic approximation was acceptable, at least for the higher vibrational frequencies, since their contributions to $\lambda_{i}$ were dominant. Indeed, as shown in Figure 4, the individual components $\lambda_{i}^{m}$ (from HR factor $S_{m}$; see also Equation (S6)) to $\lambda_{i}\;$ revealed, as is generally found for organic semiconductors, a larger contribution from vibrations above 1200 cm^−1^, most of which were associated with carbon-carbon bond stretching of the conjugated molecular core, where the largest geometry change occurred (see Figures S9 and S10). Figure 4 also shows that the active frequencies (larger $\lambda_{i}^{m}$ values) were very similar for the neutral and charged species. Compared with previous investigations on unsubstituted NDTI ($\lambda_{i}=0.263$ eV), [31] our computed$\;\lambda_{i}$ values were slightly larger on account of the role of the flexible substituents. Notably, inspection of the computed HR factor $S_{m}$ (see Figures S11 and S12) showed that a large number of low-frequency intramolecular vibrations, mostly associated with the flexible substituents, displayed unexpectedly large $S_{m}$ values that were very likely to be overestimated as a result of the anharmonic character of low-frequency vibrations. More specifically, for **α-NDTI** and **N-NDTI**, the highest HR factor was computed for frequencies at 96 cm^−1^ and 25 cm^−1^, respectively, being associated with accordion-like skeletal deformations [45] (see Figure S13).

Because vibrational frequencies below 200 cm^−1^ can be considered classical degrees of freedom at room temperature, the effective parameters collected in Table 2 and used to evaluate the charge transfer rate constants (see the Marcus-Levich-Jortner (MLJ) formulation in Equation (4)), were determined by retaining only vibrations above 200 cm^−1^. For comparison, the effective parameters computed using the entire set of vibrational contributions are collected in Table S7.

Finally, we note that the magnitudes of the computed reorganization energies, compared with the largest computed transfer integrals (never exceeding 50 meV), implied a substantial reliability of the non-adiabatic hopping mechanism ($\lambda_{i}\;$>> $V_{ij}$) [39,48].

### 2.3. Charge Transfer Rate Constants and KMC Simulations

**Figure 5:**
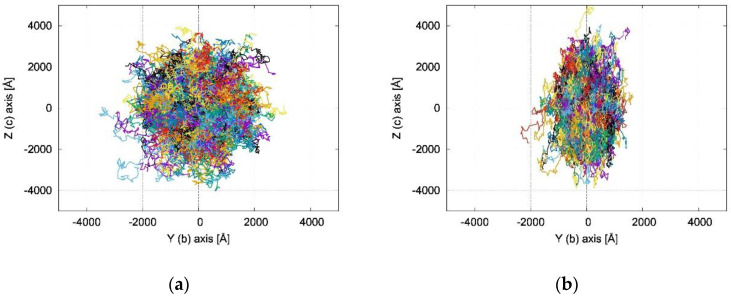
Plot of KMC trajectories (each trajectory with a different color) in the Y(≡*b*), Z(≈*c*) plane, showing the 2D character of the charge transport and a different degree of anisotropy for (**a**) **N-NDTI** *ANTI-1* and (**b**) **N-NDTI** *ANTI-2*.

The MLJ rate constants $k_{eT}$, in the absence of an applied electric field and computed for the charge transfer paths discussed in the previous section, are collected in Table 1 and Table S3. The computed values for path P1 of both NDTI derivatives were large (10^12^–10^13^ s^−1^) and similar, as was expected from the $\lambda_{i}\;$and $V_{ij}$ values. Starting from $k_{eT}$, we generated the KMC (Brownian) trajectories collected in Figure 5 and Figures S14 and S15.

**Figure 6:**
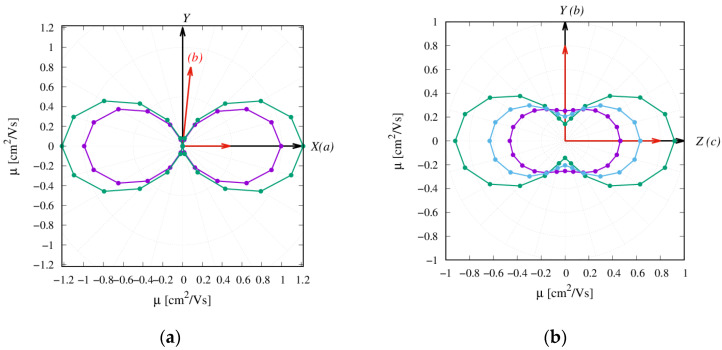
Anisotropy of charge transport from TOF KMC simulations for (**a**) **α-NDTI** and (**b**) **N-NDTI**. Purple represents *ANTI-1*, green represents *ANTI-2*, and cyan represents the *mix*.

Figure S14 confirms the 1D charge transport of **α-NDTI**, as expected from the computed transfer integrals and as previously documented for other NDTI derivatives [31]. Similarly, the columnar charge transport resulted in a remarkable anisotropy of the computed time-of-flight (TOF) charge mobilities as depicted in Figure 6a.

The KMC trajectories in Figure 5 and Figure S15 show that the charge transport in **N-NDTI** was essentially bidimensional and parallel to the *b,c* crystallographic plane as a result of non-negligible transfer integrals for paths P1, P2, and P3 (Figure 3). The *b,c* plane almost coincided with the Y,Z Cartesian plane (Figure 5), on which the trajectories’ projections were therefore the largest. In comparing **N-NDTI** *ANTI-1* and *ANTI-2*, their charge transport trajectories showed similar displacements along Z (almost coincident with *c*). For *ANTI-1*, the extension of the charge displacement in the two directions (Y,Z) was quite similar, although the P1 rate constant (along *c* ≈ Z) was larger than that of P2 (along *b* = Y) (Table 1). However, because the intermolecular distance for path P2 was larger than that of P1, the charge hopping through P2 covered a larger distance along *b* (Y) compared with that covered through P1 along *c* (≈Z). This interplay between rate constants and intermolecular distances spanned by the charge led to a modest anisotropy overall in the *b,c* (or Y,Z) plane for **N-NDTI** *ANTI-1* and a more pronounced one for *ANTI-2*. For the latter, indeed, the rate constant for jump P1 largely dominated over that of P2. Similar differences could be seen in the computed anisotropies of the TOF charge mobilities (see Figure 6b). Focusing on the three crystal structures of **N-NDTI** investigated, namely *ANTI-1*, *ANTI-2*, and the *mix*, the first (*ANTI-1*, purple in Figure 6b) showed slightly lower mobilities than the others and the lowest anisotropy. The second (*ANTI-2* in green) displayed larger and more anisotropic mobilities, and finally, the last one (*mix* in cyan) was characterized by an intermediate situation.

Overall, the computed charge mobilities from the zero field (Brownian) and TOF KMC simulations (see Table 3) indicated a similar charge transport efficiency for both NDTI derivatives, with computed values to the order of 1 cm^2^ V^−1^ s^−1^. Such similar computed mobilities were, however, in contrast with the experimental observation of a superior charge transport efficiency for **N-NDTI** vs. **α-NDTI**.

A possible explanation for the lowest observed mobility of **α-NDTI** may be related to experimental factors, such as contact resistance limitations, additional structural disorder at the single crystal level, and chemical impurities. However, in seeking intrinsic factors, we suggest here that the remarkably anisotropic charge transport of **α-NDTI** plays a relevant role. The X-ray diffraction (XRD) measurements indeed showed the *c*-axis of **α-NDTI** single crystal standing out of the OFET substrate, while the *a,b* plane was arranged parallel to the substrate. However, the calculations showed that only along the *a* direction the mobility was large. In other directions, namely in the *a,b* plane, which was parallel to the OFET substrate (compare *μ*_max_ ≈ 1 cm^2^ V^−1^ s^−1^ and *μ*_min_ ≈ 10^−6^ cm^2^ V^−1^ s^−1^ in Table 3) and in the direction perpendicular to it, the mobility reduced to almost zero. This might justify the discrepancy between the computed and experimental values, with the latter averaging the OFET mobility in the *a,b* plane (rather than only along one direction).

XRD measurements of the **N-NDTI** single crystal displayed the *a*-axis standing on the substrate, while the *b,c* plane was arranged parallel to the substrate. In contrast to **α-NDTI**, the KMC simulations showed a similar charge transport efficiency in every direction of the *b,c* plane (i.e., the substrate plane) of **N-NDTI**, and therefore, a favorable mobility is always expected in this case. Furthermore, the KMC trajectories (Figure S15) also showed a non-negligible charge transport along the *a* direction, which was relevant for the bottom gate’s top contact OFET architecture used in the experiments [49].

A second, minor discrepancy between the computed and observed results concerned the slight underestimation of the largest computed TOF mobility of **N-NDTI** (0.92 cm^2^ V^−1^ s^−1^) compared with the highest experimental mobility (1.56 cm^2^ V^−1^ s^−1^) [32]. This is somewhat unusual, since predicted mobilities are generally overestimated due to the ideal conditions underlying the models used. Several computational and experimental reasons may account for this discrepancy. Although the conditions for the non-adiabatic hopping were satisfied (for both NDTI derivatives), other factors could have limited the quantitative agreement of the computed charge mobilities, among them being the fact that MLJ formulation relies on a single effective parameter and that our KMC simulations did not include the effects of carrier-carrier interactions, a factor that may be relevant for high charge densities [49]. In this frame, charge mobility is a function of several parameters, among which is the charge density, which was not taken into account here [50,51]. We should further mention that from the experimental side, the measured OFET mobilities could be overestimated, as was suggested in previous works [52,53].

Overall, the above considerations may explain the differences between the observed and computed mobilities. Aside from these factors, dynamic disorder may also play a role in affecting the final computed charge mobility, as is discussed in the next section.

### 2.4. Influence of Dynamic Disorder

Extrinsic sources of disorder can affect carrier transport, as has been shown theoretically [54] and experimentally [55]. In addition, there is a general consensus that dynamic disorder is one of the crucial parameters governing charge transport in organic semiconductors. Generally, thermally induced fluctuations of transfer integrals limit charge transport [37,41,56], but for percolation channels characterized by small electronic couplings, they have been shown to open new hopping pathways, thereby increasing the mobility [34,57,58,59,60]. In recent works, it has been reported that the strength of the dynamic disorder is highly correlated with the gradient of the electronic couplings [56,61] with respect to the phonon modes, a parameter also known as the non-local electron-phonon coupling (or Peierls coupling) [39,40]. Further, the presence of alkyl chains may influence the dynamic disorder via modulation of electron–phonon couplings. In a recent work on alkylated dinaphthothienothiophene (DNTT) [62], it was shown that transfer integral fluctuations are prevalently dominated by a single sliding mode involving long-axis displacements between pairs of molecules. The enhancement of the thermal disorder by a such sliding mode was shown to dramatically depress the charge mobility. For this reason, the sliding mode was identified as a *killer* phonon.

In previous works, we already investigated the role of dynamic disorder in PDI and NDI derivatives [33,34,35] by running molecular dynamics (MD) simulations followed by QC evaluation of the transfer integrals. The analysis of fluctuations via a Fourier transform of the autocorrelation function of the transfer integrals [63] provided indications of the most active intermolecular phonons. Long and short molecular axis sliding motions were clearly identified as being largely responsible for the transfer integral fluctuations and, interestingly, they were demonstrated to *activate* (i.e., a phonon-assisted mechanism) additional charge transport channels in a PDI derivative [34].

**Figure 7:**
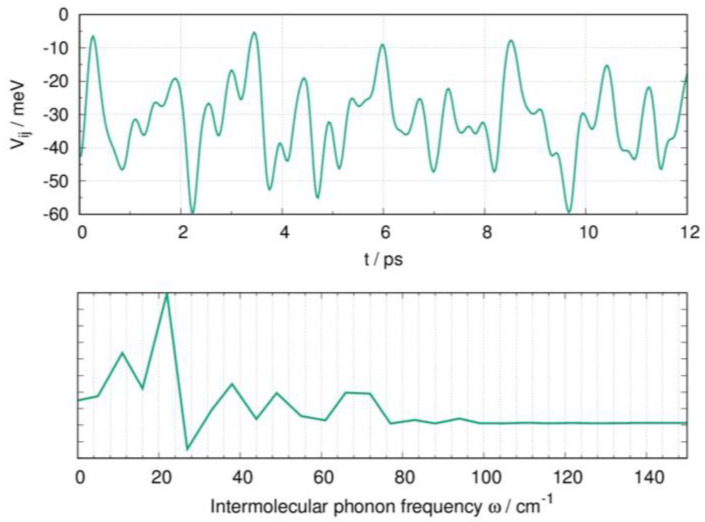
Dynamic disorder effects on the crystal of **N-NDTI** from MD simulations, showing (**top**) the fluctuations of transfer integrals $V_{ij}$ for the P1 charge transfer path, computed from snapshots of MD at 300 K and (**bottom**) a Fourier transform of the autocorrelation function of the transfer integrals, showing the activity of low-frequency intermolecular phonons.

Seeking possible evidence of phonon-assisted charge transport, we applied a similar strategy to investigate the role of thermally induced disorder in **N-NDTI** and computed the transfer integral fluctuations for the two charge pathways displaying limited efficiency (i.e., low $V_{ij}$ (P2 and P3; see Table 1)), therefore being more promising for phonon-assisted charge transport activation. The thermal fluctuations, however, were negligible for the two paths, and therefore we completed the investigation including path P1. However, for the latter, due to its large electronic coupling, the dynamic disorder effects may only be detrimental to charge transport, ultimately lowering the mobility. In Figure 7 (top), we show the computed fluctuations of the P1 transfer integrals. The standard deviation (σ) was only 11 meV, about one third of the average electronic coupling ($<V_{ij}>=$ 35 meV; see Table 1), a value smaller than what is typically found (i.e., generally the same size as the electronic couplings). The phonon frequencies, which were most active in determining the P1 transfer integral fluctuations, are shown in Figure 7 (bottom). Low-frequency intermolecular phonons (below 50 cm^−1^) were dominant, and they have been shown to be associated with sliding modes, as was reported in several previous works [34,62,64,65]. However, because the strength of the non-local electron–phonon coupling is related to σ [40,66], its modest value suggests only minor effects of the sliding modes on the **N-NDTI** charge mobility. Similar to **N-NDTI**, it can be argued that the sliding phonon modes would also be the primary lattice vibrations determining the P1 transfer integral fluctuations in **α-NDTI**.

**Figure 8:**
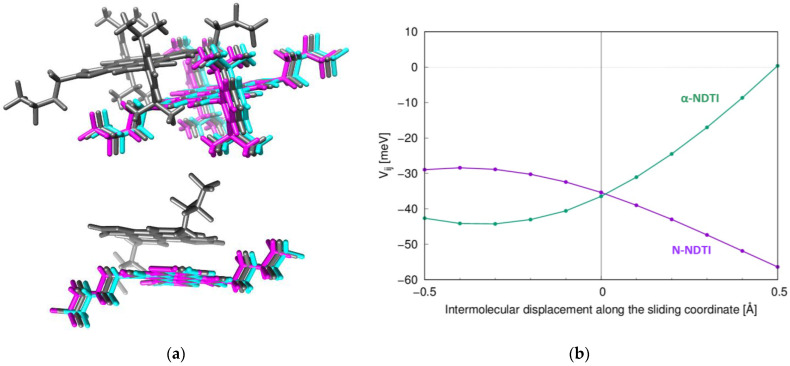
(**a**) Intermolecular sliding motion considered for (top) **α-NDTI** and (bottom) **N-NDTI**. Gray molecules represent the crystal structure of the P1 dimer. Cyan and magenta represent displaced molecules along the sliding coordinates. (**b**) Electronic coupling dependence on the sliding coordinates (**α-NDTI** in green and **N-NDTI** in purple).

Since electronic coupling fluctuations along P1 are expected to depress charge mobility (due to the large $V_{ij}$ value), it is interesting to compare **α-NDTI** and **N-NDTI**, seeking evidence for an additional mechanism accounting for the lower experimental mobility of the former. To this end, the modulations of the P1 electronic couplings were computed along the sliding coordinates represented in Figure 8a. The computed coupling dependences are collected in Figure 8b, from which the absolute value of the numerically estimated derivatives (associated to the non-local electron–phonon coupling) was 48 meV/Å for **α-NDTI** and 33 meV/Å for **N-NDTI**. The larger electron–phonon coupling for **α-NDTI** may therefore have contributed to reducing its charge transport efficiency, as was similarly shown in a recent work on alkylated DNTT [62]. Although a more detailed assessment of the entire spectrum of non-local electron–phonon couplings should be carried out for a conclusive response, the preliminary results of such a higher dependency of **α-NDTI** on thermal fluctuations than **N-NDTI** may further justify its lower observed charge mobility (Table 3), in addition to the factors discussed in the previous section.

## 3. Computational Methods and Models

The bulk charge transport was investigated according to the non-adiabatic hopping mechanism. The charge mobilities were evaluated while either assuming a Brownian motion of the charge carrier—that is, in the limit of a zero field and zero concentration—and under the effect of an electric field (see below). According to the hopping model, the relevant charge transfer event is localized on a molecular pair (dimer) formed by two neighboring molecules. The validity of this model depends on the relative magnitude of the electronic coupling $V_{ij}$ and the internal reorganization energy $\lambda_{i}$, with $V_{ij}$ required to be considerably smaller than $\lambda_{i}$ [39,48,67,68]. We verified (vide infra) that this was the case for both molecules investigated here.

The experimentally available crystal structures of the organic semiconductors were used to identify the possible charge pathways by evaluating the vector displacements of the centers of mass for the molecules surrounding a central reference site in the crystal.

While the electronic couplings were evaluated for the molecular dimers extracted from the crystal structures, calculation of the intramolecular reorganization energies was based on the determination of potential energy surfaces for neutral and charged species. The equilibrium structures were obtained from quantum chemical calculations carried out at the B3LYP/6-31G* level of theory. The nature of the stationary points determined by quantum chemical structure optimization was assessed by evaluating the vibrational frequencies at the optimized geometries. The vibrational frequencies were also employed to estimate the vibrational contributions to the intramolecular reorganization energies through the calculation of the HR parameters (see below).

### 3.1. Reorganization Energy and Electronic Couplings

The reorganization energy is composed of an intramolecular term $\lambda_{i}$ and an outer sphere contribution $\lambda_{o}$, due to the interaction with the surrounding molecules in the crystal. The former was computed either with the AP method, namely via two point determinations from each potential energy surface (neutral and charged states), or via calculations of the HR factor $S_{m}$ [67,69,70], obtained in turn within the harmonic approximation from the dimensionless displacement parameter $B_{m}$, which is generally employed to evaluate Franck–Condon (FC) vibronic progressions in electronic and photoelectronic spectra (see the SI for further details) [71,72]. The outer sphere reorganization energy $\lambda_{o}$ was assumed to be 0.01 eV according to recent determinations [46,47]. The performance of different schemes designed for efficient calculation of the intermolecular transfer integrals and site energies for pairs of molecules were reviewed recently [67,73,74,75]. In the framework of the dimer approach and one-electron approximation, the electronic coupling (charge transfer integrals) $V_{ij}=<\phi_{i}|\hat{H}|\phi_{j}>$, where $\phi_{i,j}$ are the highest occupied molecular orbital (HOMO) or LUMO orbitals of the two monomers forming the dimer (for *p*-type and *n*-type conduction, respectively), was obtained using a fragment orbital approach. Following previous studies [76,77,78,79], the protocol was based on the determination of the matrix $H_{MOB}$ in the monomer orbital basis (MOB), whose off-diagonal elements were the non-orthogonalized electronic couplings:

(1) $$H_{MOB}=C_{MON\_AOB}^{t}S_{MON\_AOB}C_{DIM\_AOB}\varepsilon_{DIM}C_{DIM\_AOB}^{t}S_{MON\_AOB}C_{MON\_AOB}$$

where $\varepsilon_{DIM}$ is the diagonal matrix of the eigenvalues associated to the molecular orbitals of the dimer, $C_{DIM\_AOB}$ is the matrix of the eigenvectors of the dimer in the atomic orbital basis (AOB), $S_{MON\_AOB}$ is the overlap matrix of the monomers in the AOB, and $C_{MON\_AOB}$ is the monomer-localized orbitals matrix. $C_{MON\_AOB}$ is, therefore, a block diagonal matrix containing the MO coefficients in the AOB from each monomer, with the off-block diagonals set to zero and the superscript *t* indicating the transpose.

The computed couplings were then transformed in an orthogonalized basis by performing a Löwdin orthogonalization:

(2) $$H_{MOB}^{⊥}=S_{DIM\_MOB}^{-\frac{1}{2}}H_{MOB}S_{DIM\_MOB}^{-\frac{1}{2}}$$

where $S_{DIM\_MOB}$ is the overlap matrix between monomer orbitals, which was obtained as follows from the MO coefficients of the monomer orbitals and the overlap of the atomic orbitals in the dimer configuration $S_{DIM\_AOB}$:

(3) $$S_{DIM\_MOB}=C_{MON\_AOB}^{t}\times S_{DIM\_AOB}\times C_{MON\_AOB}$$

For a dimer, this was conducted on the 2 × 2 $H_{MOB}$ matrix including the HOMO (or LUMO) orbitals of the two monomers [73,80]. A detailed discussion of the approximations involved in the fragment orbital approach was reported in a previous work [81].

The electronic couplings were computed at the same level of theory (B3LYP/6-31G*) adopted for geometry optimization and for the evaluation of the reorganization energies. All QC calculations were carried out with the Gaussian16 suite of programs [82].

### 3.2. Charge Transfer Rate Constants and Kinetic Monte Carlo Simulations

Because the quantum nature of the most active modes governing local electron-phonon coupling cannot be neglected, a suitable formulation of the transfer rate constants $k_{eT}$ associated with each hopping event is provided by the MLJ quantum correction of Marcus’ equation [83]:

(4) $$k_{eT}=\frac{2\pi }{\hbar }{V_{ij}}^{2}\frac{1}{\sqrt{4\pi \lambda_{o+classic}k_{B}T}}\overset{\infty }{\underset{\upsilon =0}{\sum }}\left[\exp\left(-S_{eff}\right)\frac{S_{eff}^{\upsilon }}{\upsilon !}exp\left(-\frac{{\left(\Delta G^{0}+\lambda_{o+classic}+\upsilon \hbar \omega_{eff}\right)}^{2}}{4\lambda_{o+classic}k_{B}T}\right)\right]$$

In the MLJ formulation of the charge transfer rate constants, the quantum description of the non-classical degrees of freedom is represented by a single effective mode of frequency $\omega_{eff}$ and the associated HR factor $S_{eff}$, determined from the set of computed HR factors (see the SI for additional details). Following previous work [33,34], because frequency vibrations below roughly 150–200 cm^−1^ at room temperature can be described to a good approximation in classical terms, and because of their possible anharmonicity, their contributions were not included in the evaluation of $\omega_{eff}$. The exceeding classical contributions were summed to the outer sphere reorganization energy $\lambda_{o}$, and the total contribution reads as $\lambda_{o+classic}$ in Equation (4).

The zero field (Brownian) charge mobilities were determined by computing the diffusion coefficient D with a set of KMC simulations [57,66,84,85,86,87,88] (see the SI for further details). An approximately linear dependence of the mean square displacement (MSD) ${<[r\left(t\right)-r\left(0\right)]>}^{2}$ as a function of time *t* was obtained by averaging over the subsets of 1000 KMC trajectories. The diffusion coefficient D was obtained from the fitted linear dependence of *MSD* by employing Einstein’s equation:

(5) $$D=\underset{t\to \infty }{\lim}\left(MSD/6t\right).\;$$

The charge mobility was then obtained by Einstein–Smoluchowski’s equation:

(6) $$\mu =\frac{eD}{k_{B}T}$$

In the presence of an electric field, the TOF charge mobilities were obtained with the following relation by applying an electric field F of magnitude 10^5^ V/cm:

(7) $$\mu =\frac{df}{\tau F}$$

where $d_{f}$ is the distance traveled by the charge along the F direction and τ is the time required to travel the distance $d_{f}$, which is assumed to be 50 μm. The mobility was averaged over 100 trajectories.

### 3.3. Simulation of Thermally Induced Dynamic Disorder

To assess the importance of thermal motions to the electronic couplings [64,89], we ran MD simulations combined with the QC evaluation of the charge transfer integrals. Molecular dynamics simulations were run on a 5×5×5 supercell of the crystal unit cell of **N-NDTI**. The dynamics of the system was studied with periodic boundary conditions employing the MM3 force field [90] and the Tinker code [91]. Since recent studies have shown that low-frequency intermolecular vibrations can modulate the magnitude of the electronic couplings [33,34,35,40,43,61,92,93,94,95], we froze all the intramolecular degrees of freedom (rigid body approximation) while allowing intermolecular motions. We ran a 100-ps MD simulation in the NVT ensemble and at T = 300 K using a thermal bath. The integration time step was set to 1 fs, and trajectory snapshots were saved every 30 fs. On a selected range of 12 ps, after equilibration, we evaluated the LUMO transfer integral fluctuations for the most relevant charge pathways of **N-NDTI** and determined their standard deviation.

## 4. Conclusions

We investigated the charge transport properties of **α-NDTI** and **N-NDTI,** two recently synthesized NDTI derivatives in which fluoroalkyl chains were introduced to improve the n-type character and device stability in ambient conditions.

The computed electronic couplings showed that fluoroalkyl substitution impacted the solid state organization of the two NDTI derivatives; the active charge transfer channels favored a 1D charge percolation in **α-NDTI** and induced a more isotropic charge transfer network in **N-NDTI.** For both, the intra-column (P1) electronic coupling dominated. The computed intramolecular reorganization energies were similar for **α-NDTI** and **N-NDTI** and slightly larger compared with the unsubstituted NDTI, underscoring the role of the low-frequency vibrations associated with skeletal deformations.

The similar magnitudes of the computed intra- and intermolecular parameters was reflected in the n-type mobilities, predicted to be to the order of 1 cm^2^ V^−1^ s^−1^ and comparable for **α-NDTI** and **N-NDTI**, in contrast with the experimental observation of a superior charge transport efficiency for **N-NDTI.**

Disregarding the role of impurities, contact resistance limitations, and other experimental factors, this apparent discrepancy could be reconciled by considering the striking difference in the charge mobility anisotropy. While **N-NDTI** displayed sizable charge mobilities in every direction parallel to the substrate, **α-NDTI** exhibited a prominent anisotropic character with mobility that reduced to zero in some directions parallel to the substrate. We suggested that such marked anisotropy of **α-NDTI** could justify the difference between the computed and experimental values, the latter corresponding to the average OFET mobilities.

Seeking additional sources of charge mobility degradation for **α-NDTI,** we also compared the role of dynamic disorder on the most effective charge transport path (P1) of both derivatives. Although limited to transfer integral fluctuations induced by an intermolecular sliding mode, the computed electron–phonon couplings confirmed a more detrimental effect for **α-NDTI** compared with **N-NDTI**.

The lower observed mobility of **α-NDTI** was therefore rationalized in terms of the strong anisotropic character of the charge percolation pathways, with the additional contribution of dynamic disorder effects.

## Figures and Tables

**Table 1:** Charge hopping paths (Pn) relevant for charge transport and the intermolecular distances for each dimer, inter-planar distances, and electronic couplings *V_ij_* (B3LYP/6-31G*) between the LUMO orbitals and Marcus-Levich-Jortner (MLJ) rate constants *k_eT_* computed for three crystalline structures *ANTI-1*, *ANTI-2*, and *mix* of **α-NDTI** and **N-NDTI**.

Dimer or Charge Transfer Path	Molecules Forming the Dimer	Intermolecular Distance (Å)	Interplanar Distance (Å)	*V_ij_* (LUMO) (meV)	*k_eT_ ^a^* (s^−1^)
**α-NDTI**					
P1	*ANTI-1*	6.654	3.646	36	5.62 × 10^12^
	*ANTI-2*	6.654	3.646	40	6.88 × 10^12^
	*mix*	6.654	3.646	38	
**N-NDTI**					
P1	*ANTI-1*	4.197	3.623	35	6.87 × 10^12^
	*ANTI-2*	4.197	3.623	50	1.36 × 10^13^
	*mix*	4.193–4.202	3.700–3.744	46–38	1.15 × 10^13^–7.91 × 10^12^
P2	*ANTI-1*	11.002	0.144	10	5.26 × 10^11^
	*ANTI-2*	11.002	0.144	8	3.05 × 10^11^
	*mix*	11.002–11.002	0.244–0.278	9–8	4.91 × 10^11^–3.89 × 10^11^
P3	*ANTI-1*	11.238	3.467	2	2.59 × 10^10^
	*ANTI-2*	11.238	3.467	1	5.12 × 10^9^
P4	*ANTI-1*	12.290	3.033	0	4.40 × 10^8^
	*ANTI-2*	12.290	3.033	0	1.56 × 10^8^

*^a^* From Equation (4), without an applied electric field.

**Table 2:** Intramolecular reorganization energy $\lambda_{i}$, effective frequency ω_eff_, and effective HR factor S_eff_, with contributions from intramolecular classical vibrations $\lambda_{classic}$ and from the outer sphere $\lambda_{o}$ to $\lambda_{o+classic}$ in Equation (4) for **α-NDTI** and **N-NDTI**.

	${\lambda_{i}\;}^{a}$ (eV)	ω_eff_ ^a^ (cm^−1^)	S_eff_ ^a^	${\lambda_{classic}\;}^{b}$ (eV)	${\lambda_{o}\;}^{c}$ (eV)
**α-NDTI** *ANTI-1*	0.286	920	2.51	0.032	0.01
**α-NDTI** *ANTI-2*	0.286	925	2.49	0.033	0.01
**N-NDTI** ^d^	0.294	849	2.79	0.011	0.01

^a^ Computed by excluding vibrations with frequencies lower than 200 cm^−1^. ^b^ Contribution due to intramolecular vibrations lower than 200 cm^−1^. ^c^ Value chosen according to [46,47]. ^d^ Only one value was reported in this case, since both *ANTI-1* and *ANTI-2* structures, featuring different thiophene orientations in the crystal, converged to the same single-molecule equilibrium geometry.

**Table 3:** Computed Brownian (zero field) and TOF mobilities for **α-NDTI** and **N-NDTI**.

	Zero Field (Brownian)	TOF	TOF	*Exp*
	*μ* (cm^2^ V^−^^1^ s^−1^)	*μ*_max_ (cm^2^ V^−1^ s^−1^)	*μ*_min_ (cm^2^ V^−1^ s^−1^)	*μ ^a^* (cm^2^ V^−1^ s^−1^)
**α-NDTI** *ANTI-1*	0.32	1.00 *^b^*	3 × 10^−6^ *^b^*	0.037
**α-NDTI** *ANTI-2*	0.39	1.23 *^b^*	2 × 10^−6^ *^b^*	
**N-NDTI** *ANTI-1*	0.24	0.46 *^c^*	0.25 *^c^*	1.27
**N-NDTI** *ANTI-2*	0.34	0.92 *^c^*	0.14 *^c^*	
**N-NDTI** *mix*	0.28	0.63 *^c^*	0.21 *^c^*	

*^a^* Average experimental (Exp) mobilities from [32]. *^b^* TOF mobilities computed in the X,Y plane. *^c^* TOF mobilities computed in the Z,Y plane.

## Supplementary Materials

The following are available online: Figures S1–S15 and Tables S1–S7.

## References

[1] Usta H., Facchetti A., Marks T.J. (2011). n-Channel semiconductor materials design for organic complementary circuits. Acc. Chem. Res..

[2] Newman C.R., Frisbie C.D., da Silva Filho D.A., Brédas J.-L., Ewbank P.C., Mann K.R. (2004). Introduction to drganic thin film transistors and design of n-channel organic semiconductors. Chem. Mater..

[3] Okamoto T., Kumagai S., Fukuzaki E., Ishii H., Watanabe G., Niitsu N., Annaka T., Yamagishi M., Tani Y., Sugiura H. (2020). Robust, high-performance n-type organic semiconductors. Sci. Adv..

[4] Dhar J., Salzner U., Patil S. (2017). Trends in molecular design strategies for ambient stable n-channel organic field effect transistors. J. Mater. Chem. C.

[5] Quinn J.T.E., Zhu J., Li X., Wang J., Li Y. (2017). Recent progress in the development of n-type organic semiconductors for organic field effect transistors. J. Mater. Chem. C.

[6] Wang S., Sun H., Ail U., Vagin M., Persson P.O.Å., Andreasen J.W., Thiel W., Berggren M., Crispin X., Fazzi D. (2016). Thermoelectric properties of solution-processed n-doped Ladder-type Conducting polymers. Adv. Mater..

[7] Usta H., Newman C., Chen Z., Facchetti A. (2012). Dithienocoronenediimide-based copolymers as novel ambipolar semiconductors for organic thin-film transistors. Adv. Mater..

[8] Yue W., Nikolka M., Xiao M., Sadhanala A., Nielsen C.B., White A.J.P., Chen H.-Y., Onwubiko A., Sirringhaus H., McCulloch I. (2016). Azaisoindigo conjugated polymers for high performance n-type and ambipolar thin film transistor applications. J. Mater. Chem. C.

[9] Yang J., Xiao B., Tajima K., Nakano M., Takimiya K., Tang A., Zhou E. (2017). Comparison among Perylene Diimide (PDI), Naphthalene Diimide (NDI), and Naphthodithiophene Diimide (NDTI) based n-type polymers for all-polymer solar cells application. Macromolecules.

[10] Yang J., Xiao B., Tang A., Li J., Wang X., Zhou E. (2019). Aromatic-Diimide-based n-type conjugated polymers for all–polymer solar cell applications. Adv. Mater..

[11] Samanta S.K., Song I., Yoo J.H., Oh J.H. (2018). Organic n-channel transistors based on [1]benzothieno[3,2-*b*]benzothiophene–rylene diimide donor-acceptor conjugated polymers. ACS Appl. Mater. Interfaces.

[12] Xiao C., Jiang W., Li X., Hao L., Liu C., Wang Z. (2014). Laterally Expanded Rylene Diimides with Uniform Branched Side Chains for Solution-Processed Air Stable n-Channel Thin Film Transistors. ACS Appl. Mater. Interfaces.

[13] Hecht M., Würthner F. (2021). Supramolecularly Engineered J-Aggregates Based on Perylene Bisimide Dyes. Acc. Chem. Res..

[14] Qiao Y., Guo Y., Yu C., Zhang F., Xu W., Liu Y., Zhu D. (2012). Diketopyrrolopyrrole-containing quinoidal small molecules for high-performance, air-stable, and solution-processable n-channel organic field-effect transistors. J. Am. Chem. Soc..

[15] Lin G., Qin Y., Zhang J., Guan Y.-S., Xu H., Xu W., Zhu D. (2016). Ambipolar organic field-effect transistors based on diketopyrrolopyrrole derivatives containing different π-conjugating spacers. J. Mater. Chem. C.

[16] Katz H.E., Lovinger A.J., Johnson J., Kloc C., Siegrist T., Li W., Lin Y.-Y., Dodabalapur A. (2000). A soluble and air-stable organic semiconductor with high electron mobility. Nature.

[17] Hu Y., Gao X., Di C., Yang X., Zhang F., Liu Y., Li H., Zhu D. (2011). Core-expanded Naphthalene Diimides Fused with Sulfur Heterocycles and End-Capped with Electron-Withdrawing Groups for Air-Stable Solution-Processed n-Channel Organic Thin Film Transistors. Chem. Mater..

[18] Gao X., Di C., Hu Y., Yang X., Fan H., Zhang F., Liu Y., Li H., Zhu D. (2010). Core-Expanded Naphthalene Diimides Fused with 2-(1,3-Dithiol-2-Ylidene)Malonitrile Groups for High-Performance, Ambient-Stable, Solution-Processed n-Channel Organic Thin Film Transistors. J. Am. Chem. Soc..

[19] Usta H., Risko C., Wang Z., Huang H., Deliomeroglu M.K., Zhukhovitskiy A., Facchetti A., Marks T.J. (2009). Design, synthesis, and characterization of ladder-type molecules and polymers. Air-stable, solution-processable n-channel and ambipolar semiconductors for thin-film transistors via experiment and theory. J. Am. Chem. Soc..

[20] Lee W.-Y., Oh J.H., Suraru S.-L., Chen W.-C., Würthner F., Bao Z. (2011). High-Mobility Air-Stable Solution-Shear-Processed n-Channel Organic Transistors Based on Core-Chlorinated Naphthalene Diimides. Adv. Funct. Mater..

[21] Suraru S.-L., Würthner F. (2014). Strategies for the Synthesis of Functional Naphthalene Diimides. Angew. Chem. Int. Ed..

[22] Bhosale S.V., Bhosale S.V., Bhargava S.K. (2012). Recent progress of core-substituted naphthalenediimides: Highlights from 2010. Org. Biomol. Chem..

[23] Chen Z., Zheng Y., Yan H., Facchetti A. (2009). Naphthalenedicarboximide- vs Perylenedicarboximide-based copolymers. Synthesis and semiconducting properties in bottom-gate n-channel organic transistors. J. Am. Chem. Soc..

[24] Fukutomi Y., Nakano M., Hu J., Osaka I., Takimiya K. (2013). Naphthodithiophenediimide (NDTI): Synthesis, structure, and applications. J. Am. Chem. Soc..

[25] Yue W., Gao J., Li Y., Jiang W., Di Motta S., Negri F., Wang Z. (2011). One-pot synthesis of stable NIR tetracene diimides via double cross-coupling. J. Am. Chem. Soc..

[26] Zhang F., Hu Y., Schuettfort T., Di C., Gao X., McNeill C.R., Thomsen L., Mannsfeld S.C.B., Yuan W., Sirringhaus H. (2013). Critical role of alkyl chain branching of organic semiconductors in enabling solution-processed n-channel organic thin-film Transistors with Mobility of up to 3.50 cm^2^ V^–1^ s^–1^. J. Am. Chem. Soc..

[27] Nakano M., Osaka I., Hashizume D., Takimiya K. (2015). α-Modified naphthodithiophene diimides—molecular design strategy for air-stable n-channel organic semiconductors. Chem. Mater..

[28] Takimiya K., Osaka I. (2015). Naphthodithiophenes: Emerging building blocks for organic electronics. Chem. Rec..

[29] Ran H., Chen L., Yang X., Zhang J., Zhao Z., Han R., Duan X., Hu J.Y. (2019). Arylacetylene end capped naphthodithiophene diimide (NDTI)-based semiconductors for air-stable, solution-processed n-channel organic field-effect transistors: Effect of terminal aryl groups on charge transport. Dyes Pigments.

[30] Ran H., Duan X., Zheng R., Xie F., Chen L., Zhao Z., Han R., Lei Z., Hu J.-Y. (2020). Two isomeric azulene-decorated naphthodithiophene diimide-based triads: Molecular orbital distribution controls polarity change of OFETs through connection position. ACS Appl. Mater. Interfaces.

[31] Ji L.F., Fan J.X., Zhang S.F., Ren A.M. (2017). Theoretical investigations into the charge transfer properties of thiophene α-substituted naphthodithiophene diimides: Excellent n-channel and ambipolar organic semiconductors. Phys. Chem. Chem. Phys..

[32] Fan W., Liu C., Li Y., Wang Z. (2017). Fluoroalkyl-modified naphthodithiophene diimides. Chem. Commun..

[33] Di Donato E., Fornari R.P., Di Motta S., Li Y., Wang Z., Negri F. (2010). n-Type charge transport and mobility of fluorinated perylene bisimide semiconductors. J. Phys. Chem. B.

[34] Di Motta S., Siracusa M., Negri F. (2011). Structural and thermal effects on the charge transport of core-twisted chlorinated perylene bisimide semiconductors. J. Phys. Chem. C.

[35] Canola S., Negri F. (2014). Anisotropy of the n-type charge transport and thermal effects in crystals of a fluoro-alkylated naphthalene diimide: A computational investigation. Phys. Chem. Chem. Phys..

[36] Canola S., Graham C., Pérez-Jiménez Á.J., Sancho-García J.C., Negri F. (2019). Charge transport parameters for carbon based nanohoops and donor-acceptor derivatives. Phys. Chem. Chem. Phys..

[37] Fratini S., Mayou D., Ciuchi S. (2016). The transient localization scenario for charge transport in crystalline organic materials. Adv. Funct. Mater..

[38] Troisi A. (2007). Prediction of the absolute charge mobility of molecular semiconductors: The case of rubrene. Adv. Mater..

[39] Troisi A. (2011). Charge transport in high mobility molecular semiconductors: Classical models and new theories. Chem. Soc. Rev..

[40] Coropceanu V., Sánchez-Carrera R.S., Paramonov P., Day G.M., Brédas J.-L. (2009). Interaction of charge carriers with lattice vibrations in organic molecular semiconductors: Naphthalene as a case study. J. Phys. Chem. C.

[41] Harrelson T.F., Dantanarayana V., Xie X., Koshnick C., Nai D., Fair R., Nuñez S.A., Thomas A.K., Murrey T.L., Hickner M.A. (2019). Direct probe of the nuclear modes limiting charge mobility in molecular semiconductors. Mater. Horiz..

[42] Tu Z., Yi Y., Coropceanu V., Brédas J.-L. (2018). Impact of phonon dispersion on nonlocal electron-phonon couplings in organic semiconductors: The naphthalene crystal as a case study. J. Phys. Chem. C.

[43] Di Motta S., Di Donato E., Negri F., Orlandi G., Fazzi D., Castiglioni C. (2009). Resistive molecular memories: Influence of molecular parameters on the electrical bistability. J. Am. Chem. Soc..

[44] Bronstein H., Nielsen C.B., Schroeder B.C., McCulloch I. (2020). The role of chemical design in the performance of organic semiconductors. Nat. Rev. Chem..

[45] Shimanouchi T. (1973). Local and overall vibrations of polymer chains. Pure Appl. Chem..

[46] McMahon D.P., Troisi A. (2010). Evaluation of the external reorganization energy of polyacenes. J. Phys. Chem. Lett..

[47] Norton J.E., Brédas J.-L. (2008). Polarization energies in oligoacene semiconductor crystals. J. Am. Chem. Soc..

[48] Giannini S., Carof A., Ellis M., Yang H., Ziogos O.G., Ghosh S., Blumberger J. (2019). Quantum localization and delocalization of charge carriers in organic semiconducting crystals. Nat. Commun..

[49] Li H., Brédas J.-L. (2021). Developing molecular-level models for organic field-effect transistors. Natl. Sci. Rev..

[50] Schweicher G., Garbay G., Jouclas R., Vibert F., Devaux F., Geerts Y.H. (2020). Molecular Semiconductors for Logic Operations: Dead–End or Bright Future?. Adv. Mater..

[51] Schweicher G., Olivier Y., Lemaur V., Geerts Y.H. (2014). What currently limits charge carrier mobility in crystals of molecular semiconductors?. Isr. J. Chem..

[52] Bittle E.G., Basham J.I., Jackson T.N., Jurchescu O.D., Gundlach D.J. (2016). Mobility overestimation due to gated contacts in organic field-effect transistors. Nat. Commun..

[53] Paterson A.F., Singh S., Fallon K.J., Hodsden T., Han Y., Schroeder B.C., Bronstein H., Heeney M., McCulloch I., Anthopoulos T.D. (2018). Recent progress in high-mobility organic transistors: A reality check. Adv. Mater..

[54] Ciuchi S., Fratini S. (2012). Electronic transport and quantum localization effects in organic semiconductors. Phys. Rev. B.

[55] Minder N.A., Lu S., Fratini S., Ciuchi S., Facchetti A., Morpurgo A.F. (2014). Tailoring the molecular structure to suppress extrinsic disorder in organic transistors. Adv. Mater..

[56] Nematiaram T., Troisi A. (2020). Strategies to reduce the dynamic disorder in molecular semiconductors. Mater. Horiz..

[57] Martinelli N.G., Olivier Y., Athanasopoulos S., Ruiz Delgado M.-C., Pigg K.R., da Silva Filho D., Sánchez-Carrera R.S., Venuti E., Della Valle R.G., Brédas J.-L. (2009). Influence of intermolecular vibrations on the electronic coupling in organic semiconductors: The case of anthracene and perfluoropentacene. Chemphyschem.

[58] Gosar P., Vilfan I. (1970). Phonon-assisted current in organic molecular crystals. Mol. Phys..

[59] Munn R.W., Silbey R. (1985). Theory of electronic transport in molecular crystals. III. Diffusion coefficient incorporating nonlocal linear electron–phonon coupling. J. Chem. Phys..

[60] Girlando A., Grisanti L., Masino M., Brillante A., Della Valle R.G., Venuti E. (2011). Interaction of charge carriers with lattice and molecular phonons in crystalline pentacene. J. Chem. Phys..

[61] Xie X., Santana-Bonilla A., Troisi A. (2018). Nonlocal electron–phonon coupling in prototypical molecular semiconductors from first principles. J. Chem. Theory Comput..

[62] Schweicher G., D’Avino G., Ruggiero M.T., Harkin D.J., Broch K., Venkateshvaran D., Liu G., Richard A., Ruzié C., Armstrong J. (2019). Chasing the “killer” phonon mode for the rational design of low-disorder, high-mobility molecular semiconductors. Adv. Mater..

[63] Skourtis S.S., Balabin I.A., Kawatsu T., Beratan D.N. (2005). Protein dynamics and electron transfer: Electronic decoherence and non-Condon effects. Proc. Natl. Acad. Sci. USA.

[64] Troisi A., Orlandi G. (2006). Dynamics of the intermolecular transfer integral in crystalline organic semiconductors. J. Phys. Chem. A.

[65] Troisi A., Orlandi G., Anthony J.E. (2005). Electronic interactions and thermal disorder in molecular crystals containing cofacial pentacene units. Chem. Mater..

[66] Shuai Z., Geng H., Xu W., Liao Y., André J.-M. (2014). From charge transport parameters to charge mobility in organic semiconductors through multiscale simulation. Chem. Soc. Rev..

[67] Oberhofer H., Reuter K., Blumberger J. (2017). Charge transport in molecular materials: An assessment of computational methods. Chem. Rev..

[68] Cheung D.L., Troisi A. (2008). Modelling charge transport in organic semiconductors: From quantum dynamics to soft matter. Phys. Chem. Chem. Phys..

[69] Brédas J.-L., Beljonne D., Coropceanu V., Cornil J. (2004). Charge-transfer and energy-transfer processes in π-conjugated oligomers and polymers: A molecular picture. Chem. Rev..

[70] Coropceanu V., Cornil J., da Silva Filho D.A., Olivier Y., Silbey R., Brédas J.-L. (2007). Charge transport in organic semiconductors. Chem. Rev..

[71] Negri F., Zgierski M.Z. (1995). Theoretical analysis of vibronic structure of absorption spectrum of fulvene. J. Chem. Phys..

[72] Negri F., Orlandi G. (1995). The T 1 resonance Raman spectra of 1,3,5–hexatriene and its deuterated isotopomers: An ab initio re-investigation. J. Chem. Phys..

[73] Baumeier B., Kirkpatrick J., Andrienko D. (2010). Density-functional based determination of intermolecular charge transfer properties for large-scale morphologies. Phys. Chem. Chem. Phys..

[74] Kubas A., Hoffmann F., Heck A., Oberhofer H., Elstner M., Blumberger J. (2014). Electronic couplings for molecular charge transfer: Benchmarking CDFT, FODFT, and FODFTB against high-level ab initio calculations. J. Chem. Phys..

[75] Kubas A., Gajdos F., Heck A., Oberhofer H., Elstner M., Blumberger J. (2015). Electronic couplings for molecular charge transfer: Benchmarking CDFT, FODFT and FODFTB against high-level ab initio calculations. II. Phys. Chem. Chem. Phys..

[76] Canola S., Pecoraro C., Negri F. (2016). Dimer and cluster approach for the evaluation of electronic couplings governing charge transport: Application to two pentacene polymorphs. Chem. Phys..

[77] Troisi A., Orlandi G. (2001). The hole transfer in DNA: Calculation of electron coupling between close bases. Chem. Phys. Lett..

[78] Senthilkumar K., Grozema F.C., Bickelhaupt F.M., Siebbeles L.D.A. (2003). Charge transport in columnar stacked triphenylenes: Effects of conformational fluctuations on charge transfer integrals and site energies. J. Chem. Phys..

[79] Norton J.E., Brédas J.-L. (2008). Theoretical characterization of titanyl phthalocyanine as a p-type organic semiconductor: Short intermolecular π–π interactions yield large electronic couplings and hole transport bandwidths. J. Chem. Phys..

[80] Valeev E.F., Coropceanu V., da Silva Filho D., Salman S., Brédas J.-L. (2006). Effect of electronic polarization on charge-transport parameters in molecular organic semiconductors. J. Am. Chem. Soc..

[81] Schober C., Reuter K., Oberhofer H. (2016). Critical analysis of fragment-orbital DFT schemes for the calculation of electronic coupling values. J. Chem. Phys..

[82] Frisch M.J., Trucks G.W., Schlegel H.E., Scuseria G.E., Robb M.A., Cheeseman J.R., Scalmani G., Barone V., Petersson G.A., Nakatsuji H. (2016). Gaussian 16.

[83] Barbara P.F., Meyer T.J., Ratner M.A. (1996). Contemporary Issues in Electron Transfer Research. J. Phys. Chem..

[84] Olivier Y., Lemaur V., Brédas J.L., Cornil J. (2006). Charge hopping in organic semiconductors: Influence of molecular parameters on macroscopic mobilities in model one-dimensional stacks. J. Phys. Chem. A.

[85] Schrader M., Körner C., Elschner C., Andrienko D. (2012). Charge transport in amorphous and smectic mesophases of dicyanovinyl-substituted oligothiophenes. J. Mater. Chem..

[86] Baumeier B., Stenzel O., Poelking C., Andrienko D., Schmidt V. (2012). Stochastic modeling of molecular charge transport networks. Phys. Rev. B.

[87] Marsh R.A., Groves C., Greenham N.C. (2007). A microscopic model for the behavior of nanostructured organic photovoltaic devices. J. Appl. Phys..

[88] Baumeier B., May F., Lennartz C., Andrienko D. (2012). Challenges for in silico design of organic semiconductors. J. Mater. Chem..

[89] Troisi A., Orlandi G. (2006). Charge-Transport Regime of Crystalline organic semiconductors: Diffusion limited by thermal off-diagonal electronic disorder. Phys. Rev. Lett..

[90] Allinger N.L., Yuh Y.H., Lii J.H. (1989). Molecular mechanics. The MM3 force field for hydrocarbons. 1. J. Am. Chem. Soc..

[91] Rackers J.A., Wang Z., Lu C., Laury M.L., Lagardère L., Schnieders M.J., Piquemal J.P., Ren P., Ponder J.W. (2018). Tinker 8: Software tools for molecular design. J. Chem. Theory Comput..

[92] Wang L., Li Q., Shuai Z., Chen L., Shi Q. (2010). Multiscale study of charge mobility of organic semiconductor with dynamic disorders. Phys. Chem. Chem. Phys..

[93] Sánchez-Carrera R.S., Paramonov P., Day G.M., Coropceanu V., Brédas J.-L. (2010). Interaction of charge carriers with lattice vibrations in oligoacene crystals from naphthalene to pentacene. J. Am. Chem. Soc..

[94] Vener M.V., Parashchuk O.D., Kharlanov O.G., Maslennikov D.R., Dominskiy D.I., Yu. Chernyshov I., Yu. Paraschuk D., Yu. Sosorev A. (2021). Non-local electron-phonon interaction in naphthalene diimide derivatives, its experimental probe and impact on charge-carrier mobility. Adv. Electron. Mater..

[95] Beratan D.N., Skourtis S.S., Balabin I.A., Balaeff A., Keinan S., Venkatramani R., Xiao D. (2009). Steering electrons on moving pathways. Acc. Chem. Res..

