# Supplementary material for: Impact of Fluoroalkylation on the n-Type Charge Transport of Two Naphthodithiophene Diimide Derivatives
Source: Molecules. 2021 Jul 6;26(14):4119. doi: 10.3390/molecules26144119 (PMC8307299; doi:10.3390/molecules26144119)
Supplement: Supplementary file 1 [file molecules-26-04119-s001.zip › molecules-1277234-supplementary.pdf]

Supplementary Materials

Impact of fluoro-alkylation on the n-type charge transport of two naphthodithiophene-diimide derivatives

Gaetano Ricci[1,2], Sofia Canola[1,3], Yasi Dai[1], Daniele Fazzi[4,*] and Fabrizia Negri[1,5,*]

[1] Dipartimento di Chimica "Giacomo Ciamician", Università di Bologna, Via F. Selmi, 2, 40126 Bologna, Italy

[2] Present address: Unité de Chimie Physique Théorique et Structurale & Laboratoire de Physique du Solide, Namur Institute of Structured Matter, Université de Namur, B-5000, Namur, Belgium

[3] Present address: Institute of Physics of the Czech Academy of Sciences, Cukrovarnická 10/112, CZ16200 Praha 6, Czech Republic

[4] Institut für Physikalische Chemie, Department für Chemie, Universität zu Köln, Greinstr. 4-6, D-50939 Köln,Germany

[5] INSTM, UdR Bologna, Via F. Selmi, 2, 40126, Bologna, Italy

TABLE OF CONTENTS **PAGE**

Computational details 3
Table S1 – Total absolute and orbital energies....5
Table S2 – Relevant dimers for α-NDTI charge transport 5
Table S3 – α-NDTI electronic couplings and MLJ charge transfer rate constants 6
Table S4 - Relevant dimers for N-NDTI charge transport....6
Table S5 – Absolute energies employed to evaluate λi (adiabatic potential method) 7
Table S6 - Computed λi (adiabatic potential method) 7
Table S7 – Effective parameters (MLJ rate constants) without the exclusion of classical modes 7
Figure S1 – *ANTI-1* and *ANTI-2* orientations in α-NDTI and N-NDTI crystals 8
Figure S2 – Frontier molecular orbitals of α-NDTI....9
Figure S3 – Frontier molecular orbitals of N-NDTI 10
Figure S4 – Near-neighbours and possible charge transfer paths of α-NDTI.... 11
Figure S5 – The most effective charge transfer path P1 of α-NDTI 11
Figure S6 – Orientation of molecules in the N-NDTI crystal.... 12
Figure S7 – Orbital overlap along the P1 path, in N-NDTI *ANTI-1* and *ANTI-2* crystals.... 13
Figure S8 – The N-NDTI *mix* structure 14
Figure S9 – Bond length changes upon charging α-NDTI.... 15
Figure S10 – Bond length changes upon charging N-NDTI.... 16
Figure S11 – Huang-Rhys factors for N-NDTI.... 17
Figure S12 – Huang-Rhys factors for α-NDTI 17

Figure S13 – Low frequency intra-molecular modes of α-NDTI and N-NDTI 18

Figure S14 – Charge carrier trajectories for α-NDTI 19

Figure S15– Charge carrier trajectories for N-NDTI... 20

Computational details

Dimensionless displacement parameters $\mathbf{B_m}$ and Huang Rhys factors $\mathbf{S_m}$.

The B_m and S_m parameters, assuming the harmonic approximation and neglecting Duschinsky rotation, are defined as

$$B_m = \sqrt{\frac{\omega_m}{\hbar}}\left[\boldsymbol{X}_k - \boldsymbol{X}_j\right]\boldsymbol{M}^{1/2}\boldsymbol{L}_m(k) \qquad \text{(S1)}$$

$$S_m = \frac{1}{2}{B_m}^2 \qquad \text{(S2)}$$

where $\boldsymbol{X}_{k,j}$ is the 3N dimensional vector of the equilibrium Cartesian coordinates of the k, jth state (here the pristine and oxidized/reduced molecular states), $\boldsymbol{M}$ is the ***3N×3N*** diagonal matrix of atomic masses and $\boldsymbol{L}_m(k)$ is the ***3N*** dimensional vector describing the mth normal coordinate of the kth state in terms of mass weighted Cartesian coordinates.

Effective parameters in MLJ formulation.

In the MLJ rate constant formulation:

$$k_{eT} = \frac{2\pi}{\hbar}{V_{ij}}^2\frac{1}{\sqrt{4\pi\lambda_{o+classic}k_BT}}\sum_{v=0}^{\infty}\left[\exp\left(-S_{eff}\right)\frac{S_{eff}^{v}}{v!}exp\left(-\frac{\left(\Delta G^0 + \lambda_{o+classic} + v\hbar\omega_{eff}\right)^2}{4\lambda_{o+classic}k_BT}\right)\right] \qquad \text{(S3)}$$

the effective vibrational frequency ω_{eff}, was determined summing over all vibrational frequencies above 200 cm^{-1} (non-classical vibrations)

$$\omega_{eff} = \frac{\sum_{m(\omega_m > 200cm^{-1})}\omega_m S_m}{\sum_{n(\omega_n > 200cm^{-1})}S_n} \qquad \text{(S4)}$$

and the associated effective HR factor, S_{eff}, was determined as

$$S_{eff} = \lambda_i/\hbar\omega_{eff} \qquad \text{(S5)}$$

$$\lambda_i = \sum\nolimits_{m(\omega_m > 200cm^{-1})}\left(\hbar\omega_m S_m\right) = \sum\nolimits_{m(\omega_m > 200cm^{-1})}\left(\lambda_i^m\right) \qquad \text{(S6)}$$

Note that the total intra-molecular reorganization energy computed by the adiabatic potential method can be recovered by eq. (S6) summing over all λ_i^m contributions including also those corresponding to frequencies below 200 cm^{-1}.

The outer sphere contribution was assumed to be 0.01 eV. ΔG^0 is the free energy associated with the self-exchange reaction ($M_N + M_C \longrightarrow M_C + M_N$ with M_N the molecule in the neutral state and M_C the molecule in the charge state) which in the frozen-core approximation can be written as the energy difference between the molecular orbitals localized on the molecules involved in the charge transfer (in this case LUMO). Since this energy difference is equal to zero for a self-exchange reaction, $\Delta G^0 = -e\boldsymbol{F}\cdot\boldsymbol{d}$, where $\boldsymbol{F}$ is the electric field vector and $\boldsymbol{d}$ the distance vector between the centres of mass of the molecules composing the dimer.

Kinetic Monte Carlo simulations.

In each KMC simulation, a single charge carrier was let move on the crystal by hopping events occurring in a dimer. The probability associated with a hopping event which moves the charge carrier from site i to site j, P_j, was determined by MLJ rate constant as $P_j = k_j / \sum_n k_n$, where n runs over all possible paths for a charge localized on a given molecular unit in the crystal. The time associated with a single hopping event is given by $-ln\, X / k_{TOT}$, where X is a random number uniformly distributed between 0 and 1 and $k_{TOT} = \sum_n k_n$. The distance associated with the hopping is the distance between the centres of mass of the molecules involved in the charge transfer. A list of possible neighbours is available and the trajectory is advanced by choosing a random number r uniformly distributed between 0 and 1 and selecting the j-th neighbour such that $\sum_n^{j-1} P_n < r \leq \sum_n^{j} P_n$.

Table S1 – Total absolute and orbital energies

Table S1. Absolute energies, frontier orbital energies and H/L energy gaps of **α-NDTI** and **N-NDTI** molecular structures extracted from crystal structure and at their optimized geometry (B3LYP/6-31G*).

	Absolute energy [a.u.]	HOMO energy [eV]	LUMO energy [eV]	ΔE LUMO-HOMO [eV]
Crystal				
**α-NDTI** *ANTI-1*	-4930.387539	-6.29	-3.89	2.40
**α-NDTI** *ANTI-2*	-4930.377615	-6.37	-3.82	2.55
Optimized				
**α-NDTI** *ANTI-1*	-4931.012184	-6.50	-3.93	2.57
**α-NDTI** *ANTI-2*	-4931.011819	-6.50	-3.94	2.57
Crystal				
**N-NDTI** *ANTI-1*	-3600.066631	-6.29	-3.77	2.52
**N-NDTI** *ANTI-2*	-3600.046489	-6.30	-3.69	2.61
Optimized				
**N-NDTI** *ANTI-1*	-3600.219944	-6.31	-3.76	2.55
**N-NDTI** *ANTI-2*	-3600.219935	-6.31	-3.76	2.55

Table S2 – Relevant dimers for α-NDTI charge transport

Table S2. Crystal of **α-NDTI**: centre of mass distances for dimers involved in charge transport, vectorial components and identification with crystal axes. See also Figure S2.

Dimer	Intermolecular distance (Å)	X (Å)	Y (Å)	Z (Å)	Crystal axis
P1	6.654	6.654	0	0	***a***
P1	6.654	-6.654	0	0	
P2	13.569	-1.404	-13.496	0	***b***
P2	13.569	1.404	13.496	0	
P3	14.481	5.250	-13.496	0	
P3	14.481	-5.250	13.496	0	
P4	14.640	-2.854	-4.442	-13.655	***c***
P4	14.640	2.854	4.442	13.655	
P5	15.719	8.058	13.496	0	
P5	15.719	-8.058	-13.496	0	
P6	16.448	1.449	-9.054	13.655	
P6	16.448	-1.449	9.054	-13.655	

Table S3 – α-NDTI electronic couplings and MLJ charge transfer rate constants

Table S3. Centre of mass distances for dimers / charge hopping paths involved in charge transport, interplanar distances, electronic couplings (B3LYP/6-31G*) between LUMO orbitals and MLJ rate constants computed for three crystalline structures: **α-NDTI** *ANTI-1*, *ANTI-2* and *mix* (along the P1 path).

Dimer / charge hopping path	Inter-molecular distance [Å]	Molecular plane distance [Å]	V_{ij}^{LUMO} [meV]	k_{eT} [s^{-1}]
**α-NDTI** *ANTI-1*				
P1	6.654	3.646	36	$5.62 \cdot 10^{12}$
P2	13.569	7.097	0.029	$3.58 \cdot 10^{6}$
P3	14.481	10.738	0.004	$6.81 \cdot 10^{4}$
P4	14.640	9.232	0.017	$1.23 \cdot 10^{6}$
P5	15.719	3.458	0.032	$6.81 \cdot 10^{4}$
P6	16.448	2.131	0.032	$6.81 \cdot 10^{4}$
**α-NDTI** *ANTI-2*				
P1	6.654	3.646	40	$6.88 \cdot 10^{12}$
P2	13.569	7.097	0.021	$1.90 \cdot 10^{6}$
P3	14.481	10.738	0.005	$1.08 \cdot 10^{5}$
P4	14.640	9.232	0.022	$2.08 \cdot 10^{6}$
P5	15.719	3.458	0.002	$1.72 \cdot 10^{4}$
P6	16.448	2.131	0.010	$4.30 \cdot 10^{5}$
**α-NDTI** *mix*				
P1	6.654	3.646	38	/

Table S4 - Relevant dimers for N-NDTI charge transport

Table S4. Crystal of **N-NDTI**: centre of mass distances for dimers involved in charge transport, vectorial components and identification with crystal axes. See also Figure S4 and Figure 3.

Dimer	Intermolecular distance (Å)	X (Å)	Y (Å)	Z (Å)	Crystal axis
P1	4.197	-0.227	0.562	4.153	≈***c***
P1	4.197	0.227	0.562	-4.153	
P2	11.002	0	-11.002	0	***b***
P2	11.002	0	11.002	0	
P3	11.238	0.227	-10.440	-4.153	
P3	11.238	-0.227	-10.440	4.153	
P4	12.290	0.227	11.564	-4.153	
P4	12.290	-0.227	11.564	4.153	

Table S5 – Absolute energies employed to evaluate λ_i (adiabatic potential method)

Table S5. Absolute energies (B3LYP/6-31G*) of the neutral species at the neutral geometry E_n^{geo-n}, of the charged species at the charged geometry E_a^{geo-a}, of the neutral species at the charged geometry E_n^{geo-a} and of the charged species at the neutral geometry E_a^{geo-n} of **α-NDTI** (two conformers determined by geometry optimization of *ANTI*-1 and *ANTI-2* crystal structures) and **N-NDTI**.

	E_n^{geo-n} [a.u.]	E_a^{geo-a} [a.u.]	E_n^{geo-a} [a.u.]	E_a^{geo-n} [a.u.]
**α-NDTI**				
ANTI-1	-4931.012184	-4931.119766	-4931.006368	-4931.113835
ANTI-2	-4931.011819	-4931.119512	-4931.006014	-4931.113592
**N-NDTI**	-3600.219944	-3600.314781	-3600.214365	-3600.309147

Table S6 - Computed λ_i (adiabatic potential method)

Table S6. Intra-molecular reorganization energies λ_i of **α-NDTI** (two conformers) and **N-NDTI** evaluated with the adiabatic potential method at B3LYP/6-31G* level.

	$\lambda_i^n = E_n^{geo-a} - E_n^{geo-n}$ [eV]	$\lambda_i^a = E_a^{geo-n} - E_a^{geo-n}$ [eV]	$\lambda_i = \lambda_i^n + \lambda_i^a$ [eV]
**α-NDTI**			
ANTI-1	0.158	0.161	0.319
ANTI-2	0.158	0.161	0.319
**N-NDTI**	0.152	0.153	0.305

Table S7 – Effective parameters (MLJ rate constants) without the exclusion of classical modes

Table S7: Reorganization energies λ_i, effective parameters (frequencies ω_{eff}, HR factors S_{eff}) and contributions from intra-molecular classical vibrations $\lambda_{classic}$, for **α-NDTI** and **N-NDTI**, determined including the entire set of computed molecular vibrations.

	λ_i [eV]	ω_{eff} [cm^{-1}]	S_{eff}	$\lambda_{classic}$ [eV]	λ_o [eV]
**α-NDTI** *ANTI-1*	0.318	445	5.778	0	0.01
**α-NDTI** *ANTI-2*	0.319	427	6.017	0	0.01
**N-NDTI**	0.305	468	5.253	0	0.01

Figure S1 – *ANTI-1* **and** *ANTI-2* **orientations in α-NDTI and N-NDTI crystals**

**α-NDTI** *ANTI-1*	**α-NDTI** *ANTI-2*	**N-NDTI** *ANTI-1*	**N-NDTI** *ANTI-2*
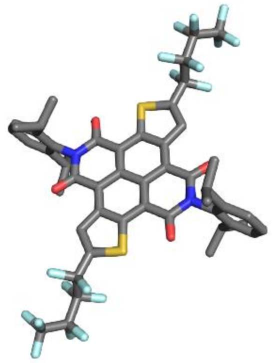	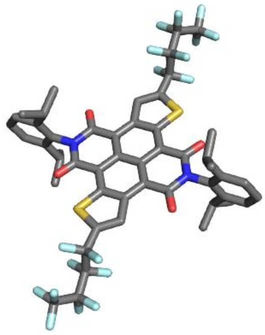	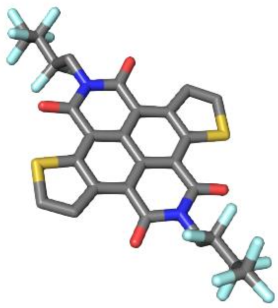	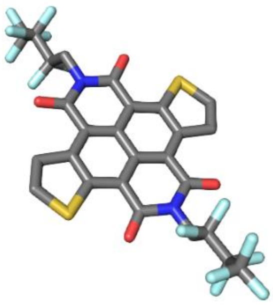

Figure S1. Crystallographic disorder: the two structures of **α-NDTI** and **N-NDTI** characterized by a different orientation of thiophene groups.

Figure S2 – Frontier molecular orbitals of α-NDTI

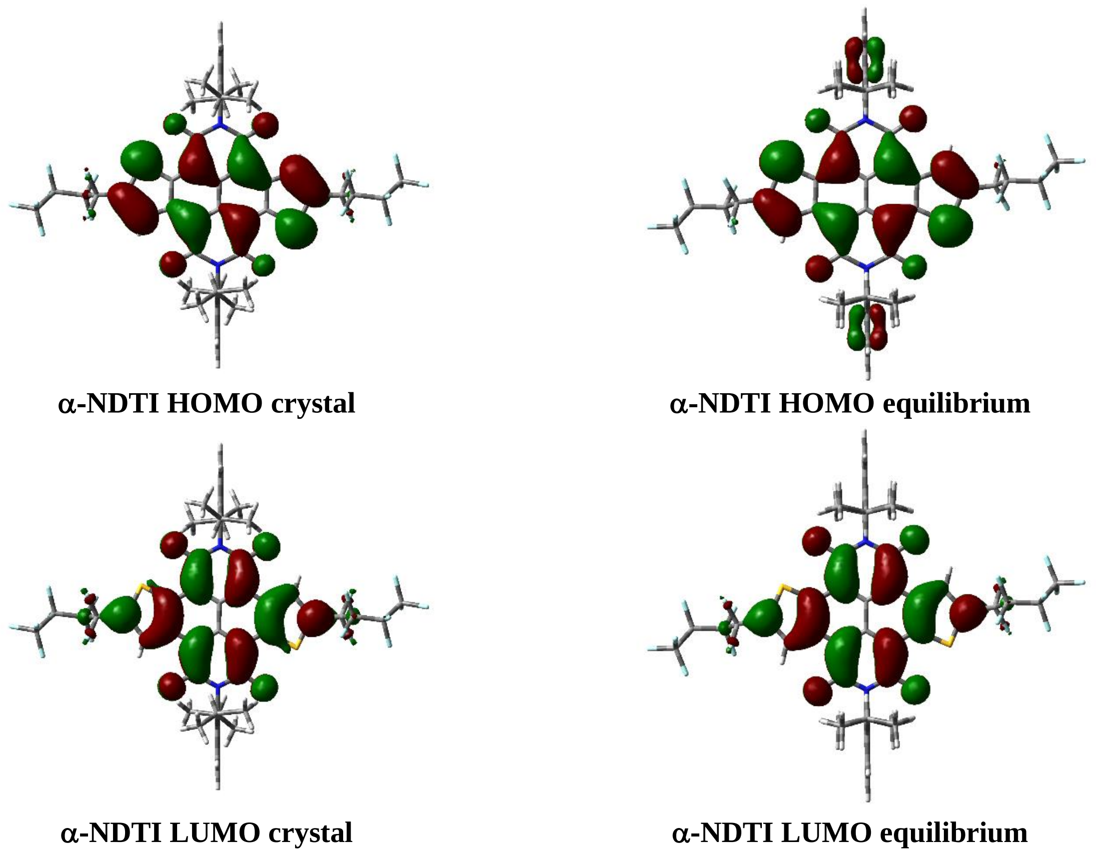

Figure S2. HOMO and LUMO orbitals of **α-NDTI** *ANTI-1* at the crystal geometry (left) and at the B3LYP/6-31G* computed equilibrium geometry (right).

Figure S3 – Frontier molecular orbitals of N-NDTI

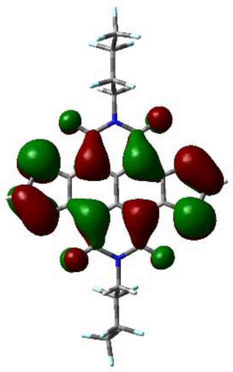

N-NDTI HOMO crystal

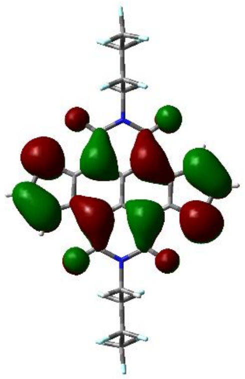

N-NDTI HOMO equilibrium

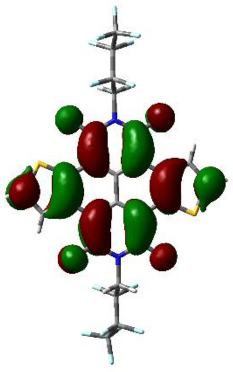

N-NDTI LUMO crystal

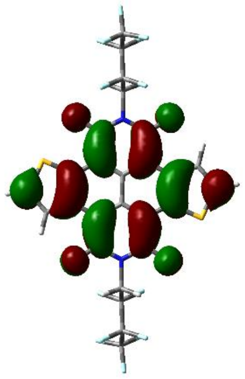

N-NDTI LUMO equilibrium

Figure S3: HOMO and LUMO orbitals of **N-NDTI** *ANTI-1* at the crystal geometry (left) and at the B3LYP/6-31G* computed equilibrium geometry (right).

Figure S4 – Near-neighbours and possible charge transfer paths of α-NDTI

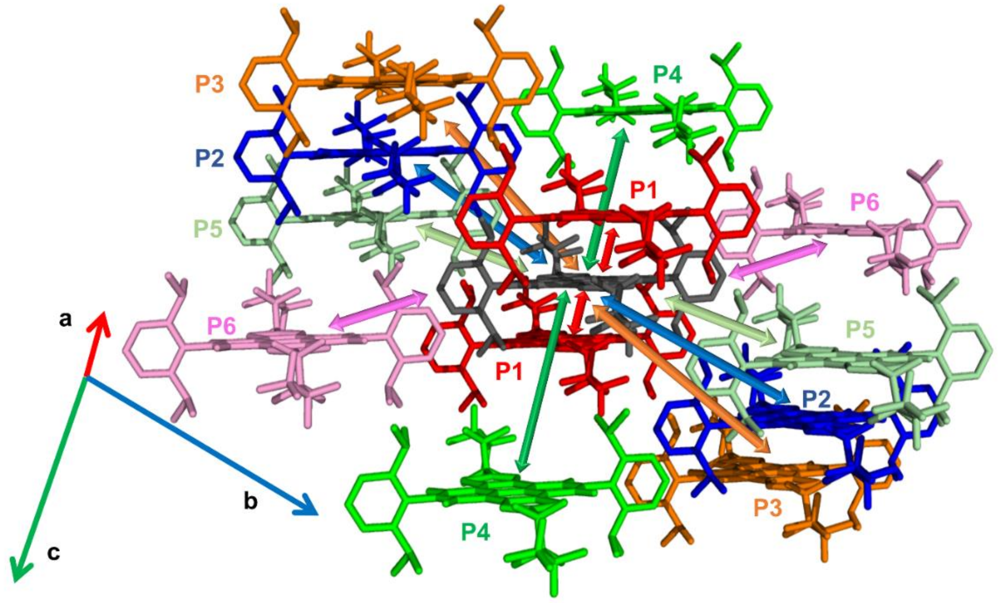

Figure S4. Crystal of **α-NDTI**: The possible charge transport paths taking as reference the central molecule (black). Only P1 is associated with a non-negligible electronic coupling.

Figure S5 – The most effective charge transfer path P1 of α-NDTI

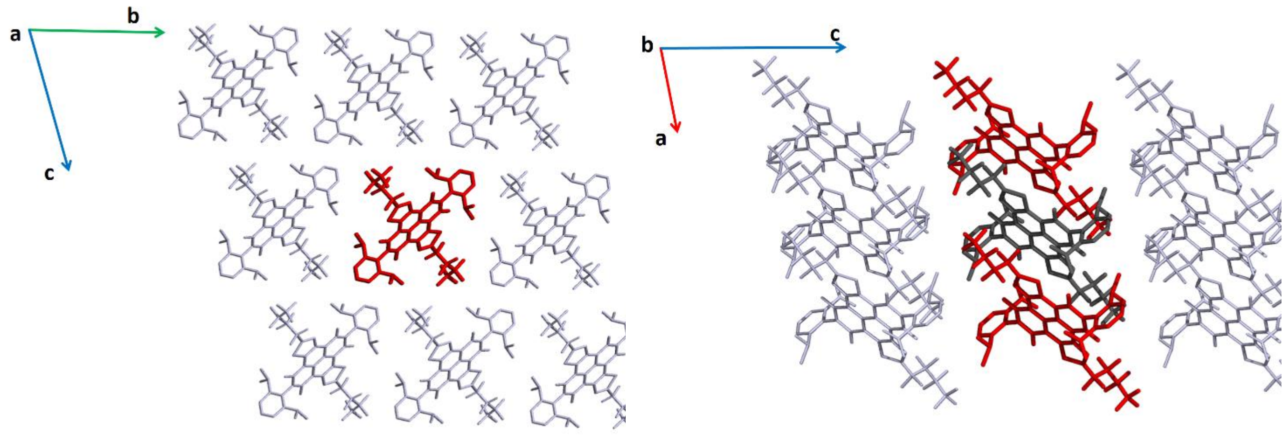

Figure S5. Crystal of **α-NDTI**: view of the P1 charge transfer path along the *a* and *b* crystal axes.

Figure S6 – Orientation of molecules in the N-NDTI crystal

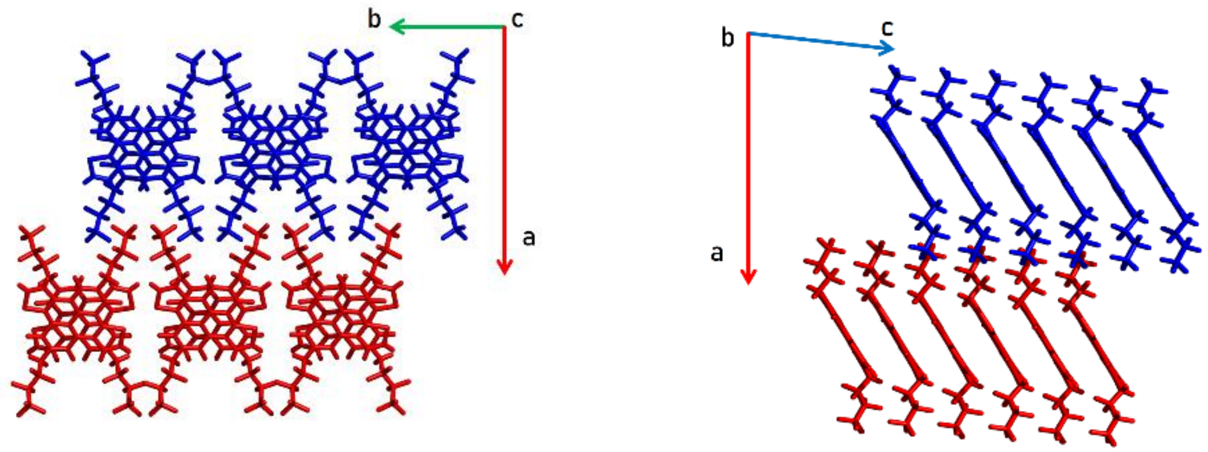

Figure S6. Crystal structure of **N-NDTI** showing layers of molecules arranged along the *c* axis and parallel to the *ac* plane. Views along the *c* and *b* crystal axes. Charge transfer integrals from the blue to the red layers are almost negligible owing to the large distance between molecules and negligible orbital overlap.

Figure S7 – Orbital overlap along the P1 path, in N-NDTI *ANTI-1* and *ANTI-2* crystals

(a)

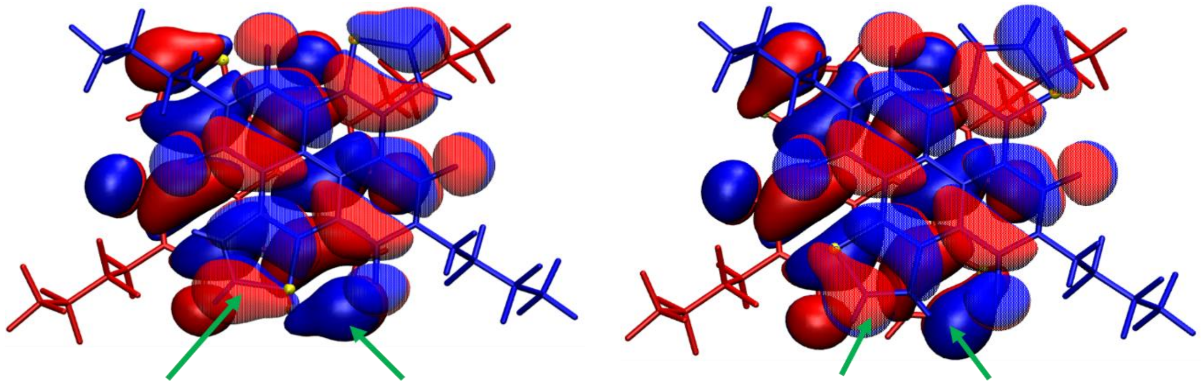

N-NDTI *ANTI-1* **N-NDTI *ANTI-2***

(b)

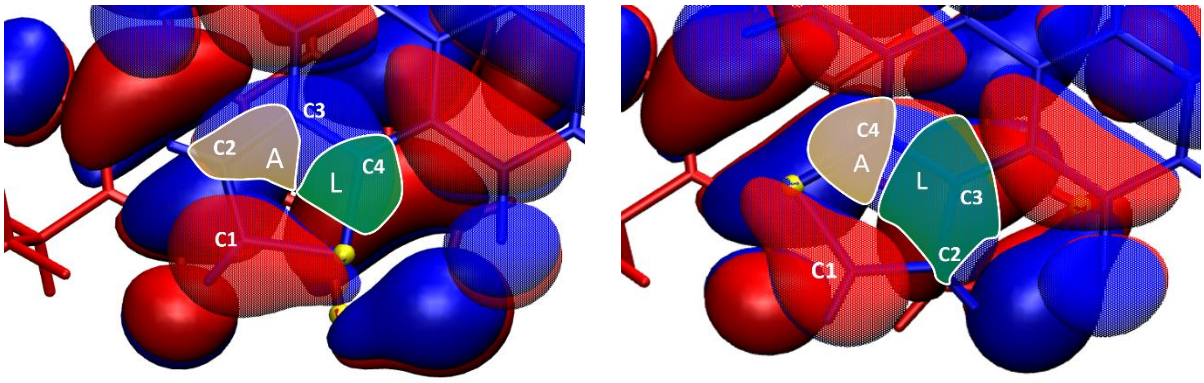

Figure S7. LUMO orbital overlap along the P1 charge transfer path of **N-NDTI.** The structure of the two molecules forming a P1 dimer are drawn in red and blue. (a) **N-NDTI** *ANTI-1* (left) and **N-NDTI** *ANTI-2* (right); (b) zoom view of the thiophene moieties indicated by green arrows in (a). C1, C2, C3 and C4 indicate the molecular moiety participating to major changes in LUMO overlaps in turn determining different computed electronic couplings for *ANTI-1* (smaller overall overlap, V_{ij}=35 meV) and *ANTI-2* (larger overall overlap, V_{ij}=50 meV) crystals. Yellow surfaces (A label) indicate regions of **'antibonding'** overlap, while green surfaces (L label) indicate regions of '**bonding**' overlap.

Figure S8 – The N-NDTI *mix* structure

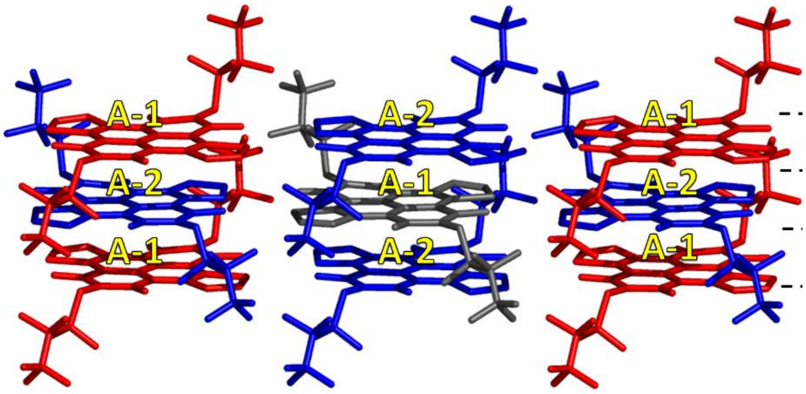

Figure S8: The **N-NDTI** *mix* crystal including both thiophene orientations *ANTI-1* (A-1) and *ANTI-2* (A-2) in equal proportions.

Figure S9 – Bond length changes upon charging α-NDTI

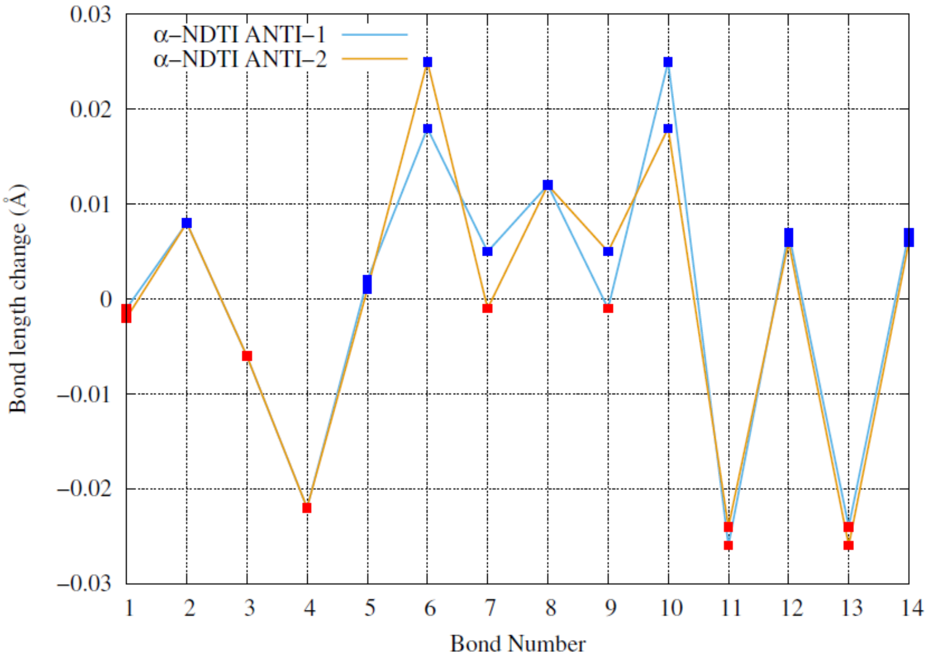

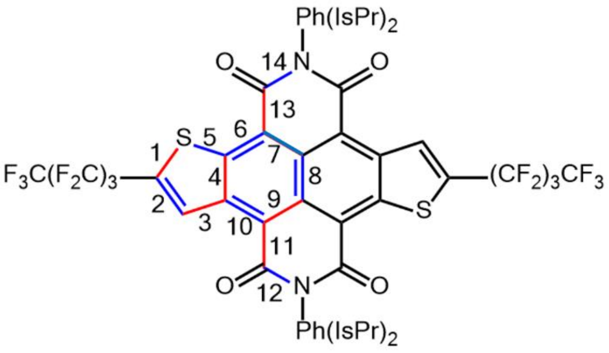

α-NDTI *ANTI-1*

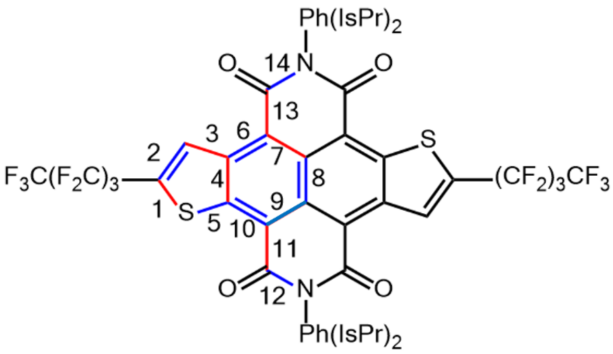

α-NDTI *ANTI-2*

Figure S9. **α-NDTI** computed bond length (BL) changes upon charging (BL(anion) - BL(neutral)) (B3LYP/6-31G*) for **α-NDTI** *ANTI-1* (cyan) and *ANTI-2* (orange).

Figure S10 – Bond length changes upon charging N-NDTI

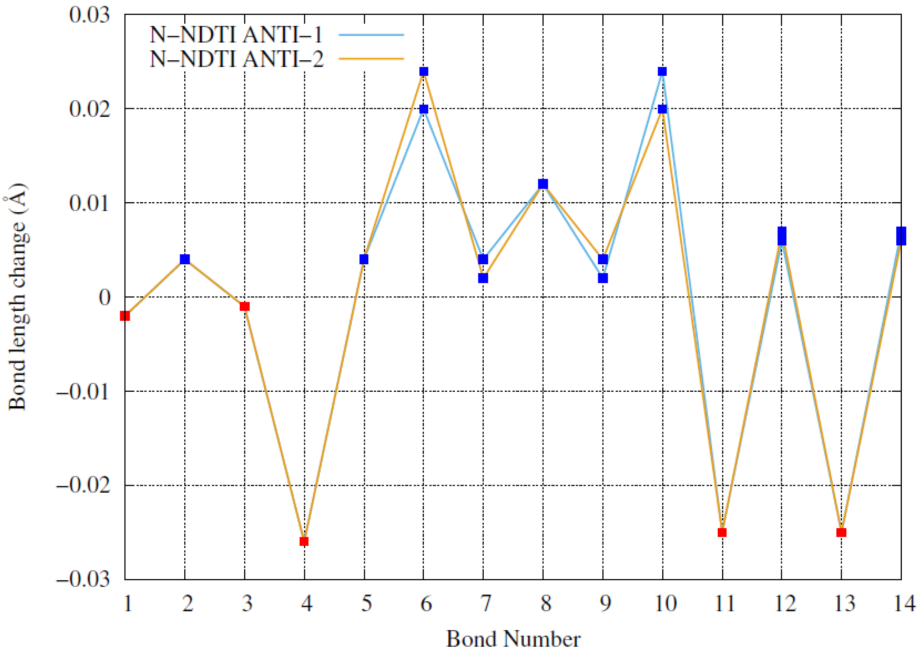

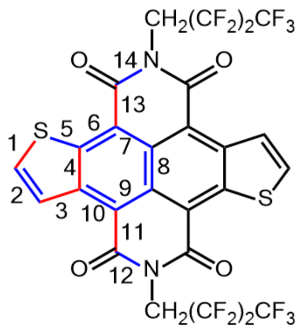

N-NDTI *ANTI-1*

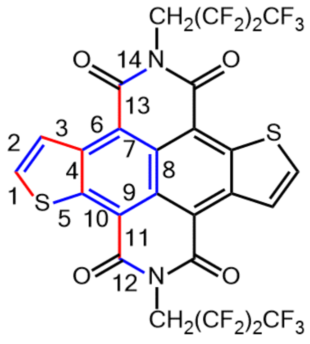

N-NDTI *ANTI-2*

Figure S10. **N-NDTI** computed bond length (BL) changes upon charging (BL(anion) - BL(neutral)) (B3LYP/6-31G*) for **N-NDTI** *ANTI-1* (cyan) and *ANTI-2* (orange).

Figure S11 – Huang-Rhys factors for N-NDTI

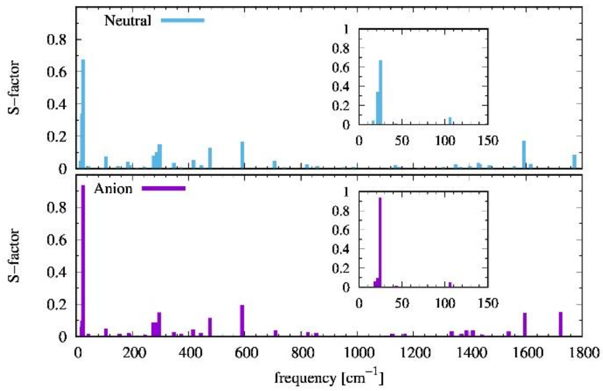

Figure S11. HR factors S_m (B3LYP/6-31G*) associated with the vibrational modes *m* of neutral and charged species of **N-NDTI**. The graph shows that largest HR factor is computed for a vibrational frequency below 50 cm^{-1}.

Figure S12 – Huang-Rhys factors for α-NDTI

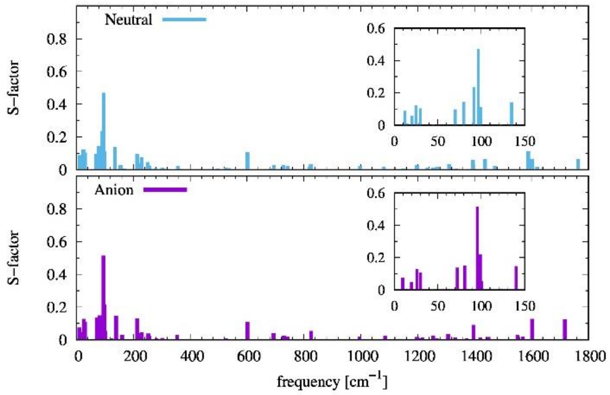

α-NDTI *ANTI-1*

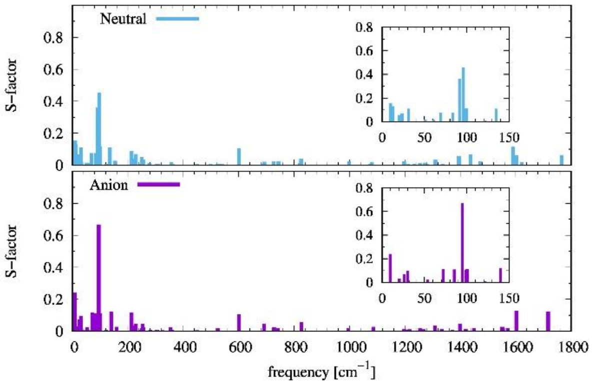

α-NDTI *ANTI-2*

Figure S12. **α-NDTI**: HR factors S_m (B3LYP/6-31G*) associated with the vibrational modes *m* of neutral and charged species of *ANTI-1* and *ANTI-2*. For both conformers, the graph shows that the largest HR factor is computed for a vibrational frequency below 100 cm^{-1}.

Figure S13 – Low frequency intra-molecular modes of α-NDTI and N-NDTI

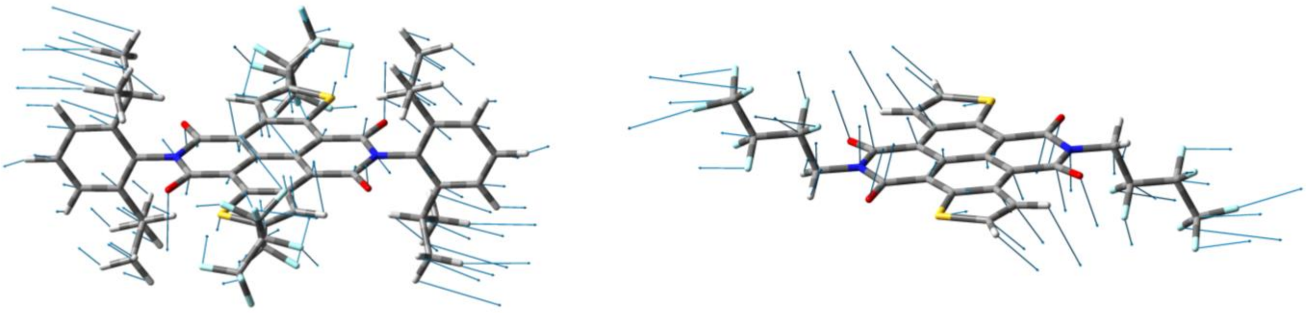

Figure S13. Visualization of the accordion-like normal modes of **α-NDTI** (left) at 96 cm^{-1} and of **N-NDTI** (right) at 25 cm^{-1} associated with large HR factors.

Figure S14 – Charge carrier trajectories for α-NDTI

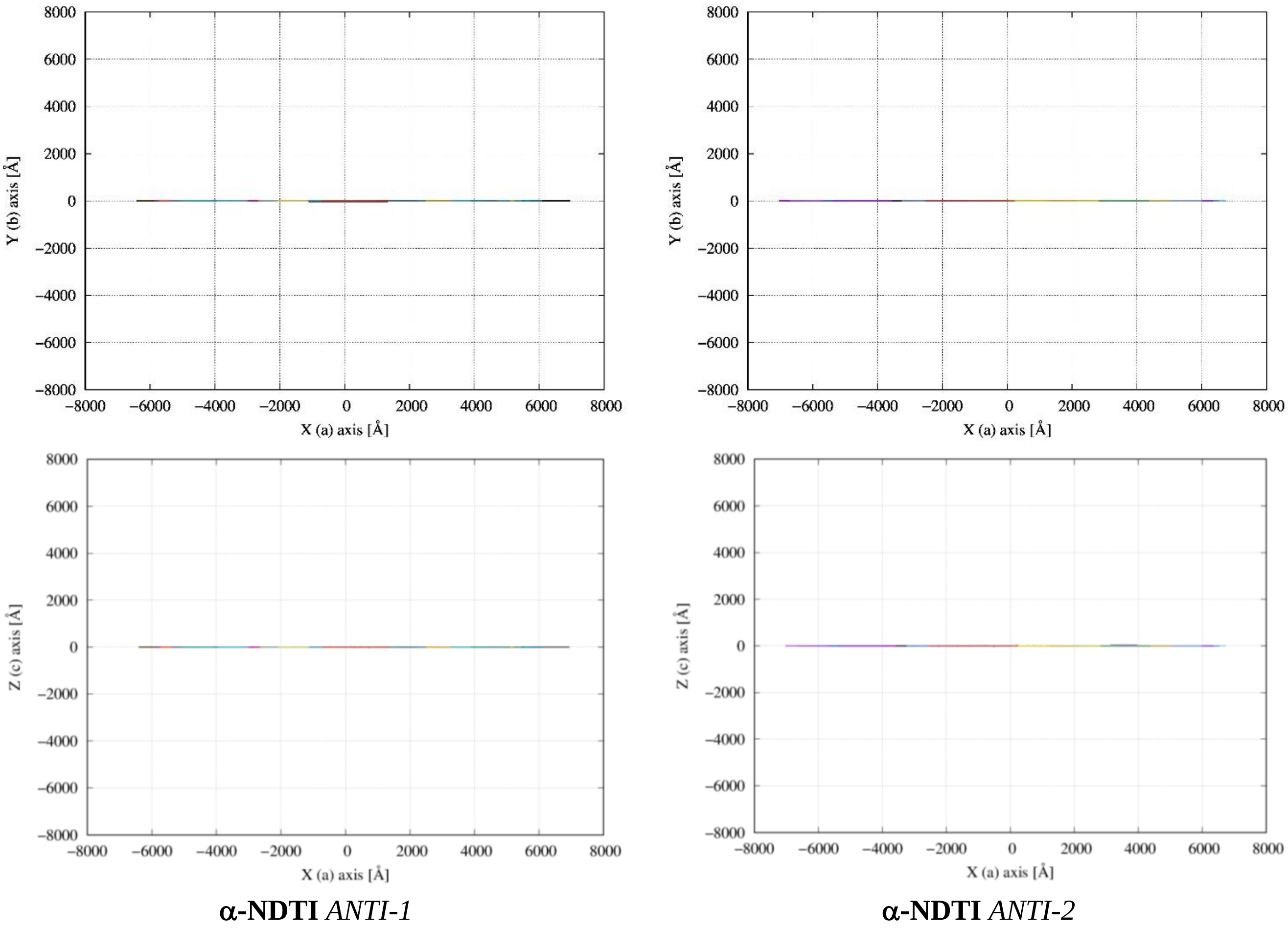

Figure S14: KMC brownian trajectories of the charge carrier computed for **α-NDTI** *ANTI-1* and *ANTI-2* and projected on (top) the X,Y plane (*a,b* crystallographic plane) and (bottom) the X,Z plane (*a,c* crystallographic plane). For both crystal structures the charge transport is 1D along the *a* axis.

Figure S15– Charge carrier trajectories for N-NDTI

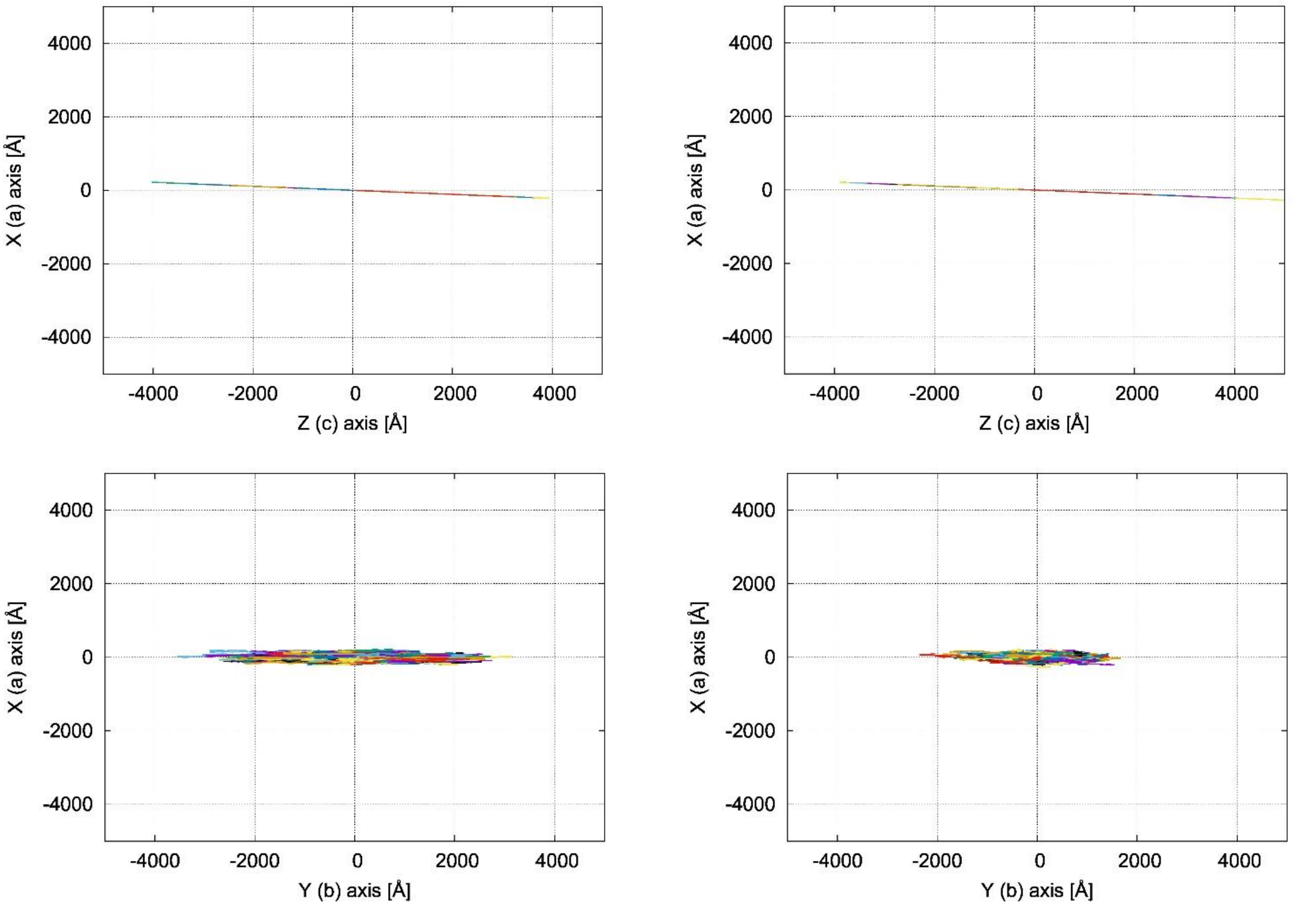

N-NDTI *ANTI-1* **N-NDTI** *ANTI-2*

Figure S15. KMC brownian trajectories of the charge carrier computed for **N-NDTI** *ANTI-1* and *ANTI-2* and projected on: (top) the X,Z plane (*a,c* crystallographic plane) and (bottom) the X,Y plane (*a,b* crystallographic plane). For both crystal structures the charge transport is bidimensional in the *b,c* plane.
